# Does model type influence the effectiveness of combined action observation and motor imagery training for novices learning an Ankle Pick takedown?

**DOI:** 10.3389/fpsyg.2025.1596660

**Published:** 2025-06-17

**Authors:** Samantha Chye, Ashika Chembila Valappil, Ryan Knight, Andrew Greene, David Shearer, Cornelia Frank, Ceri Diss, Adam Bruton

**Affiliations:** ^1^School of Life and Health Sciences, University of Roehampton, London, United Kingdom; ^2^Faculty of Life Sciences and Education, University of South Wales, Cardiff, United Kingdom; ^3^Human Movement Science Group, Sports Science, Faculty of Human and Health Sciences, University of Bremen, Bremen, Germany; ^4^Department of Sport, Health and Exercise Sciences, Brunel University of London, Uxbridge, United Kingdom

**Keywords:** motor imagery during action observation, action simulation, movement kinematics, mental representation, self-efficacy

## Abstract

**Introduction:**

Combined action observation and motor imagery (AOMI) training has been shown to facilitate motor skill performance, but limited research has explored its effectiveness on motor learning and factors that may moderate the effects of the intervention. This study examined the influence of model type on the effectiveness of AOMI training for novices learning an Ankle Pick takedown.

**Methods:**

Twenty-eight novice participants (*M* = 28.07 ± 7.29 years) were randomly assigned to a control condition (*n* = 8), or to AOMI training that displayed footage of a self-model (AOMI_SELF_; *n* = 10) or other-model (AOMI_OTHER_; *n* = 10). All training conditions included physical practice. A motor learning design incorporating pre-test (Day 1), acquisition (Days 2–6), post-test (Day 7), and retention-test (Day 14) was utilized. Motor skill performance, self-efficacy and mental representation structures were recorded as measures of learning.

**Results:**

There were no significant differences between the training conditions across all twelve kinematics measures of motor skill performance. Self-efficacy scores increased for all training conditions over time. Both the AOMI_OTHER_ and Control conditions led to improved functional changes in mental representation structures while the structures for the AOMI_SELF_ condition became less similar to the reference structure over time.

**Discussion:**

Collectively, the largely null findings (*n* = 13, 92.86%) suggest that physical practice has the strongest influence on motor adaptations for this complex motor skill at these early stages of learning. However, the findings also suggest model type may be an important factor for novices using AOMI training. It is recommended that future research explores alternative modeling approaches, such as mixed-modeling incorporating both self- and other- footage, when designing AOMI interventions for sport.

## Highlights

- Combined action observation and motor imagery (AOMI) improves motor skill performance.
- AOMI training did not facilitate learning of an Ankle Pick takedown in novices.
- AOMI displaying an other-model led to mental representations becoming more functional.
- AOMI displaying a self-model led to mental representations becoming less functional.
- AOMI training combining self- and other-models may be most effective for learning.

## Introduction

Action observation (AO) and motor imagery (MI) are two forms of motor simulation that have been shown to activate similar regions of the brain as motor execution (see Hardwick et al., 2018 for a meta-analysis). This has led to extensive research into these forms of motor simulation as training interventions whereby AO training involves the deliberate and structured observation of oneself or another individual performing the target movement (Neuman and Gray, 2013) and MI training involves the internal generation of visual and kinesthetic imagery involved in movement execution (MacIntyre et al., 2013). Both AO and MI have demonstrated positive effects on motor skill performance (see Ashford et al., 2006; Simonsmeier et al., 2021; Toth et al., 2020 for relevant meta-analyses). Since the Vogt et al. (2013) review, researchers have extensively explored the use of combined action observation and motor imagery (AOMI) as a training intervention, which involves a person systematically observing a target movement while simultaneously imagining the physiological sensations and kinesthetic experiences associated with that movement (Eaves et al., 2016). A recent meta-analysis (Chye et al., 2022) demonstrated that AOMI can significantly enhance both behavioral and neurophysiological outcomes compared to control and action observation (AO) conditions, supporting the use of AOMI training as an intervention to improve motor skill performance, and potentially learning.

Multiple theoretical accounts have proposed potential mechanisms underlying the movement benefits associated with AOMI training. Neurophysiological accounts suggest that AOMI activates motor regions of the brain to a greater extent than AO or MI training, which leads to improvements in motor execution through increased functional connectivity across these regions (see Eaves et al., 2016; Meers et al., 2020 for such accounts). From a cognitive perspective, Frank et al. (2020, 2024) and Kim et al. (2017) drew from the Cognitive Action Architecture Approach (CAA-A) to propose that AOMI can facilitate motor skill learning through the structuring of (quasi)action effects (i.e., perceptual-cognitive scaffolding). This process is thought to promote the development of more accurate and functional mental representations, which guide movement execution more comprehensively than AO or MI alone (Frank et al., 2020; Wright et al., 2022). More specifically, AOMI training supports the development of more comprehensive mental representations by refining sequencing and timing elements of mental representations through presenting visual movement information (i.e., actual action effects) through AO, and developing sensory elements of mental representations through the cognitive generation of visual and kinesthetic aspects of the movement (i.e. quasi-action effects) through MI (Frank et al., 2024; Kim et al., 2017; Wright et al., 2022). Finally, from a social psychological perspective, it has been suggested that AOMI training will improve self-efficacy, as a key self-regulatory process that underpins motor skill learning, to a greater extent than AO or MI training (Shearer et al., 2020; Wright et al., 2022). This is based on the proposition that AOMI has the capacity to provide a learner with the two strongest sources of self-efficacy (i.e., mastery experiences through MI and vicarious experiences through AO) (Bandura, 1997).

AOMI training studies have demonstrated acute benefits to motor skill performance (e.g., Marshall et al., 2020; Romano-Smith et al., 2018; Scott et al., 2018) but less is known about the longer-term, more permanent changes in motor skill proficiency (i.e., motor learning) associated with this intervention. Studies have started to investigate the efficacy of AOMI training as an intervention for learning motor skills in healthy (Binks et al., 2023a; Chye et al., 2024; Frank et al., 2023; Kim et al., 2022) and clinical populations (Binks et al., 2023b; Scott et al., 2023). For example, in a healthy population, Binks et al. (2023a) found that AOMI training significantly improved mean movement execution times for a cup-stacking task compared to AO, MI and control conditions over time. Similarly, in a clinical population, Scott et al. (2023) demonstrated that an AOMI intervention significantly improved both task completion times and movement techniques of children with developmental coordination disorder (DCD) when performing activities of daily living (e.g., shoelace tying, cutlery use, shirt buttoning). A significantly larger percentage of children in the AOMI group also successfully learnt the shoelace tying skill compared to the control condition. Collectively, these studies provide initial support for the facilitative effect of AOMI on motor learning across different populations, motor skills, and intervention periods. Given the limited body of evidence, there is still a need to better understand how AOMI training can be tailored to optimize motor learning effects. McNeill et al.'s (2020) Motor Simulation and Performance Model (MSPM) draws from empirical findings (e.g., Gatti et al., 2013; Kim et al., 2017) to suggest that AO is especially effective in the early stages of motor learning. As a result, the authors suggest that AO might be more impactful for novice learners due to the useful visual information from the modeled content without relying heavily on the learner's level of ability to update internal representations of the movement through AO. As such, the literature on AO training provides an ideal reference point for identifying methodological factors that may optimize the effects of AOMI training on motor learning outcomes.

According to Bandura's (1986), AO training is effective for motor skill learning because watching a model provides useful information about the motor skill, which helps to guide the observer's subsequent performance of the skill. This places importance on the content being modeled, and the characteristics of the model, as noted in the “What” and “Who” components of the Applied Model for the Use of Observation (AMUO; Ste-Marie et al., 2012, 2020). In terms of the content being modeled, differentiations have been made based on the skill level of the model, and subsequent accuracy of the motor skill being demonstrated. The majority of AO training studies have used skilled models who perform the motor skill successfully (e.g., Benjaminse et al., 2018; D'Innocenzo et al., 2016; Ghobadi et al., 2013). However, unskilled models that display variable and/or erroneous execution of the motor skill (e.g., Domuracki et al., 2015; Grierson et al., 2012; Shafizadeh et al., 2013), as well as learning and coping models (i.e., models that depict content that progresses toward expert-like performance) (e.g., Hebert, 2018; Welsher and Grierson, 2017), have also been compared in the AO literature (Ste-Marie et al., 2020). These various types of models likely benefit novice learners through different means. For example, skilled models are proposed to help the observer refine their cognitive representation of the motor skill by demonstrating the optimal characteristics of performing the skill (Frank et al., 2023). Conversely, unskilled, learning, and coping models may increase cognitive engagement during the modeling process by providing the observer with more opportunities to engage in error detection and strategy correction that can be applied to their future performances of the motor skill (Pollock and Lee, 1992; Rohbanfard and Proteau, 2011). There is mixed support for using the different model types for motor skill learning via AO training (see Ste-Marie et al., 2012), with studies showing that AO training incorporating either skilled and unskilled models facilitate (e.g. Barzouka et al., 2015; Hebert, 2018) or has limited impact (e.g., Al-Abood et al., 2001; Blandin et al., 1999) on learning in novice populations.

It is important to consider who is being modeled during AO training, as the similarity between the model and the observer may moderate the learning process (Bandura, 1986, 1997). According to the model-observer similarity hypothesis (Schunk, 1987), learners will be more willing to pay attention to a model that they perceive to have a greater similarity to themselves (Bandura, 1971; Maccoby and Wilson, 1957), leading to greater gains in self-efficacy and a faster rate of learning. Many studies demonstrate benefits for motor learning after AO training that utilizes self-modeling, where the novice learner acts as her/his own model (e.g., Clark and Ste-Marie, 2007; Giannousi et al., 2017), and other-modeling, where the novice learner watches another individual performing the motor skill that is being learned (e.g., Andrieux and Proteau, 2016; Weiss et al., 1998). The improved motor learning outcomes reported for both self- and other-modeling suggest an optimal model type may not exist for AO training in novice learners. Indeed, a few studies directly comparing the two model types in AO training of novice samples have reported that self-models are more effective compared to other-models (e.g., Clark and Ste-Marie, 2007; Dowrick and Raeburn, 1995; Starek and McCullagh, 1999), while others show no such difference in motor learning outcomes for the two model types (e.g., Barzouka et al., 2007; Emmen et al., 1985).

Despite recommendations for rigorous testing of different intervention design considerations to advance the AOMI training literature (Wright et al., 2022), only a few studies have directly investigated potential moderating factors when attempting to improve motor skill performance or learning through AOMI training (Chye et al., 2022). Most AOMI training studies have employed other-modeling (i.e., AOMI_OTHER_ training), with few studies exploring the use of self-modeling (i.e., AOMI_SELF_ training) for this intervention (Wright et al., 2022). While model type was not a moderator explored in Chye et al.'s (2024) meta-analysis, it is worthwhile noting that nearly all studies (*n* = 14/15 [93.3%]) used AOMI_OTHER_ training. As a result, the medium to large positive effects of AOMI on movement outcomes compared to control conditions (*d* = 0.67) can only be confidently associated with this type of AOMI training. In agreement with this assertion, prolonged bouts of AOMI_OTHER_ training benefits various movement outcomes, including force production (Di Rienzo et al., 2019; Scott et al., 2018), dart throwing accuracy (Romano-Smith et al., 2018, 2019), and rehabilitative outcomes (e.g. Bek et al., 2019; Marusic et al., 2018). The effects of AOMI_OTHER_ training are maintained irrespective of the skill level of the model, with improved movement outcomes reported for unskilled (Kawasaki et al., 2018), intermediate (Romano-Smith et al., 2018), and skilled models (Chye et al., 2024).

To-date, only four studies have investigated the effects of AOMI_SELF_ training on movement outcomes. McNeill et al. (2021) directly compared the effects of AOMI_SELF_ training, where skilled golfers viewed their own performance on a putting task, to AOMI_OTHER_ training, where skilled golfers viewed a skilled peer's performance on a putting task, on the putting performance of skilled golfers. Results showed no significant differences in putting accuracy or precision between conditions, but did show improved putter kinematics for the AOMI_SELF_ training condition, suggesting this type of AOMI training may facilitate error detection for one's own movements. Fujiwara et al. (2021) similarly compared the effects of AOMI_SELF_ training with AOMI_OTHER_ training, whereby participants either observed a video of their own hand or another person's hand performing a fine motor task using chopsticks. Results showed no significant differences in performance between the groups, but did show that participants had significantly higher MI vividness in the AOMI_SELF_ training group compared to the AOMI_OTHER_ training group. Aoyama et al. (2020) adopted a feedforward modeling approach where they manipulated the difficulty of the task being observed during AOMI_SELF_ training, such that participants watched their own performance on a novel ball rotation task at normal-difficulty (1x speed), moderate-difficulty (1.5x speed), or high-difficulty (2.5x speed). The authors found that the moderate-difficulty AOMI_SELF_ training resulted in a higher improvement rate of the ball rotation time compared to the control condition, with no such effects for the normal- or high-difficulty AOMI_SELF_ training. Finally, using an alternative feedforward approach in virtual reality for whole-body movements, Frank et al. (2023) investigated the effects of unskilled AOMI_SELF_ training, where novices observed their own movements mapped onto a 3D avatar representing themselves, compared to feedforward skilled AOMI_SELF_ training, where novices observed a skilled performer's movements mapped onto a 3D avatar representing themselves, when learning a bodyweight squat movement. There were no differences in self-efficacy scores between groups, but the feedforward skilled AOMI_SELF_ training condition demonstrated less errors in motor performance and showed more functional cognitive representations at retention compared to the unskilled AOMI_SELF_ training condition.

Given AOMI training incorporating each model type has reported positive movement outcomes, albeit with limited research investigating the effectiveness of AOMI_SELF_ training, the current study aimed to examine the influence of model type on the effectiveness of AOMI training for novices learning a complex motor skill in sport. Specifically, we compared novices' learning of an Ankle Pick takedown after 5 days of AOMI_SELF_, AOMI_OTHER_ or Control training. A novice population was selected as they naturally exhibit greater variability and adaptability in their performance (Marineau et al., 2024), thus offering a clearer window into how motor skills may develop through a motor learning intervention. The model used in the AOMI training conditions was displayed as a skeleton-like avatar of a human as per Chye et al. (2024). This allowed us to manipulate the movement characteristics by presenting either the novice's or skilled performer's movements whilst controlling the visual characteristics by presenting the same skeleton (see e.g., Frank et al., 2023). Research indicates that self-motion recognition is primarily dependent on kinematic information rather than body shape or visual appearance, allowing individuals to identify their own movements even when represented by simplified avatars (Cook et al., 2012; Thaler et al., 2018). This ability appears to be particularly pronounced for novel or infrequent actions, such as complex motor skills being learned for the first time (McDonnell et al., 2009). Thus, the use of a skeleton-like avatar in this study aligns with evidence that individuals can effectively recognize and engage with their own movement patterns based on kinematic cues alone.

Learning was inferred by recording biomechanical kinematic markers underpinning successful movements as a measure of motor skill performance, task-specific self-efficacy through a self-report questionnaire, and mental representation structures using structural dimensional analysis of mental representation (SDA-M; Schack, 2012). All three outcome measures were recorded immediately pre- and post-intervention, as well as after a one-week retention period. Based on the positive movement outcomes associated with both types of AOMI training, it was predicted that the AOMI training conditions would have positive effects on the learning measures compared to the Control training condition. Drawing from the different learning benefits proposed for self- and other-models from AO literature (Ste-Marie et al., 2012, 2020), it was hypothesized that AOMI_OTHER_ training would improve motor skill performance and mental representation structure to a greater extent than AOMI_SELF_ training due to the provision of effective movement patterns fostering adaptation and better understanding of the motor skill being learned (Frank et al., 2023; Ste-Marie et al., 2020). Based on the theoretical perspective of the model-observer similarity hypothesis (Schunk, 1987), it was hypothesized that AOMI_SELF_ training would increase novice learners' self-efficacy beliefs to a greater extent than AOMI_OTHER_ training through reinforcing the positive mastery experiences gained at this early stage of learning (Bandura, 1997; Wright et al., 2022).

## Methods

### Study design

**Figure 1:**
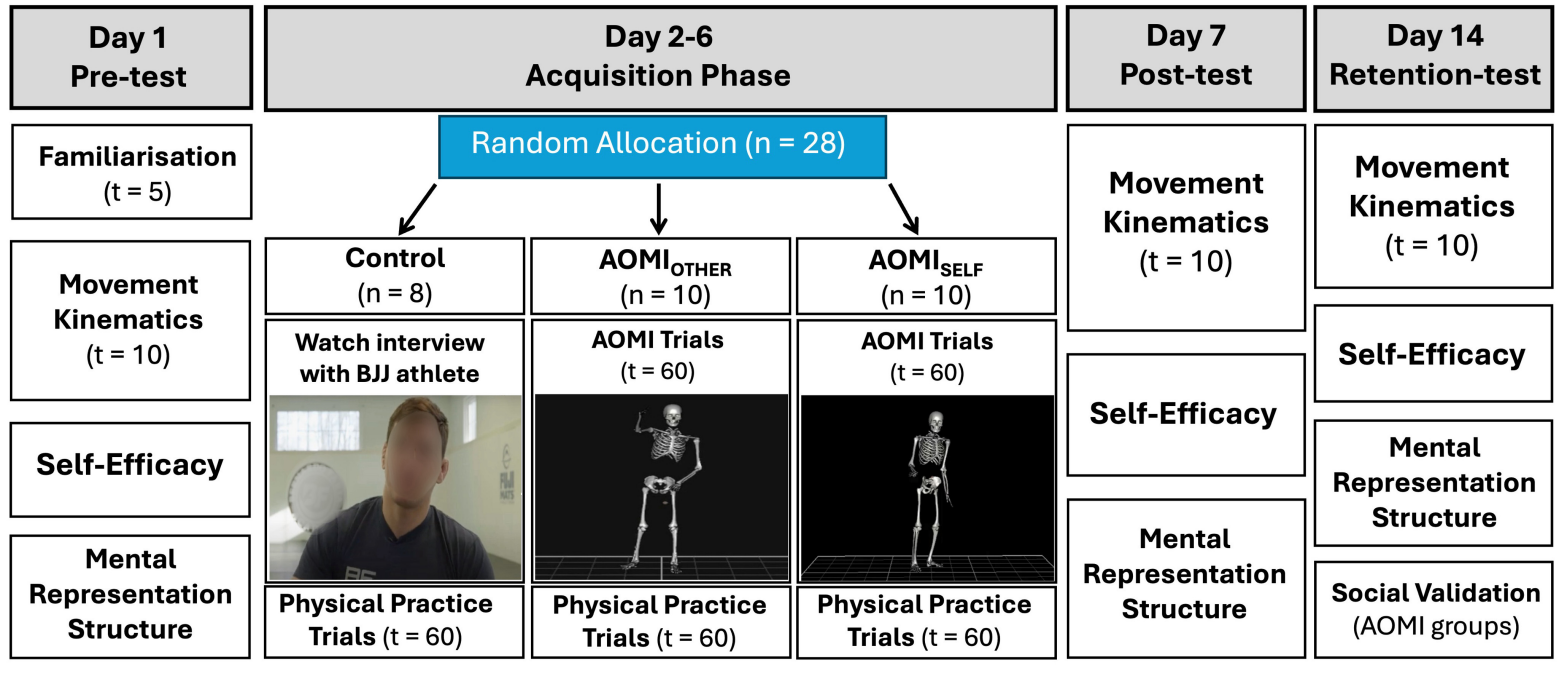
Experimental procedures across all training conditions. On Day 1, participants became familiar with the Ankle Pick takedown movement before completing a pre-test. During acquisition across Days 2–6, all participants engaged in a total of 20-min non-physical practice based on their allocated training condition alongside 300 trials of physical practice of the shadow Ankle Pick takedown movement without an opponent. On Day 7, participants returned to the laboratory for a second time and completed a post-test. On Day 14, participants returned to the laboratory for a third time and completed a retention-test followed by a social validation questionnaire and semi-structured interview.

The study was conducted in accordance with ethical guidelines and the study approval was granted from the University of Roehampton Ethical Committee. All study materials are stored as Supplementary files on the Open Science Framework (https://osf.io/3qgty/). The study employed a motor learning design (see Figure 1 for an overview of the study protocol) that incorporated pre-test (Day 1), acquisition (Days 2–6), post-test (Day 7), and 1-week delayed retention-test (Day 14) phases. The between-subject factor was training condition (AOMI_SELF_ vs. AOMI_OTHER_ vs. Control) and the within-subject factor was test phase (pre-test vs. post-test vs. retention-test). The dependent variables recorded to measure learning were motor skill performance, self-efficacy, and mental representation structures.

### Participants

Twenty-eight participants (*n* = 17 male, *n* = 11 female; *M* = 28.07 ± 7.29 years) took part in the experiment. An *a priori* power analysis based on a medium-large positive effect of AOMI interventions on movement outcomes (*d* = 0.67, *f* = 0.34; Chye et al., 2022) was used to determine the study sample size via G^*^Power (Faul et al., 2007); *F* tests, repeated measures, within-between interaction, for a Type I error probability of 0.05, a Type II error probability of 0.90 (Cohen, 1992). A study sample of twenty-seven participants was required to achieve adequate power, but thirty participants were recruited to account for potential dropout (resultant *f* = 0.31). Two participants withdrew from the study prior to completing all study phases, resulting in a final sample size of twenty-eight participants (resultant *f* = 0.33). This final sample size is in line with a recent study examining the influence of AO perspective on AOMI training effects for motor learning in novice grappling athletes (Chye et al., 2024). Participants were classified as novices in Brazilian jiu-jitsu and grappling sports (< 6 months experience or < 1-year experience at least 10 years prior to her/his study start date). Participants were screened for imagery ability using the Vividness of Movement Imagery Questionnaire 2 (VMIQ-2; Roberts et al., 2008). VMIQ-2 scores indicated that participants were able to generate moderately clear and vivid internal imagery (*M* = 1.80 ± 0.69), and clear and vivid external (*M* = 2.57 ± 0.86) and kinesthetic imagery (*M* = 2.02 ± 0.86).

### Motor skill

**Figure 2:**
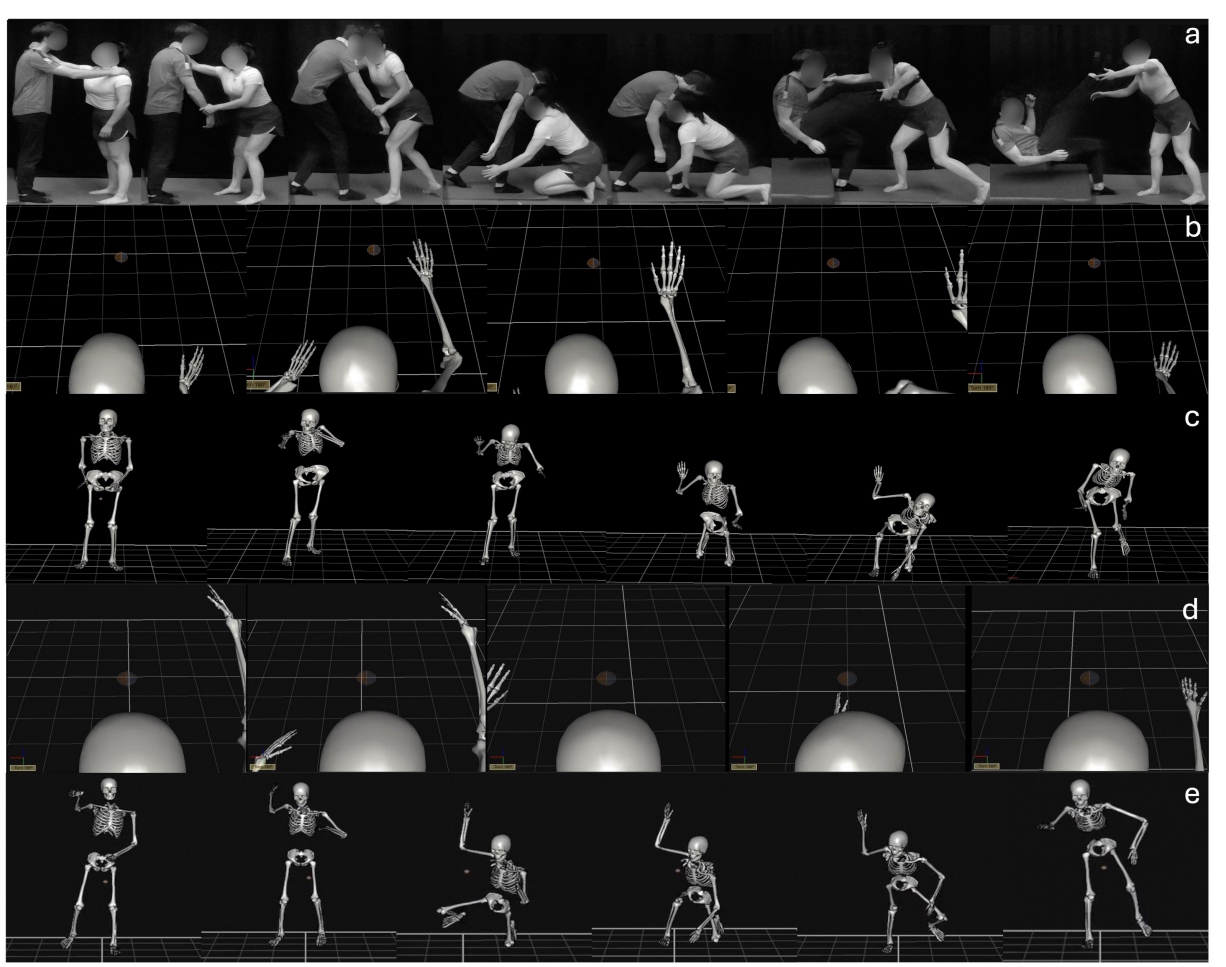
A visual depiction of **(a)** a skilled grappling athlete demonstrating the Ankle Pick takedown with an opponent, **(b)** exemplar egocentric perspective and **(c)** allocentric perceptive skeleton-like avatar video footage for one of the novice learners allocated to the AOMI_SELF_ training condition, **(d)** the egocentric perspective and **(e)** allocentric perspective skeleton-like avatar video footage for a skilled performer adopted in the AOMI_OTHER_ training condition.

The motor skill to be learned in this study was an Ankle Pick takedown (see Figure 2a for a series of images depicting the motor skill being performed by a skilled grappling athlete on an opponent). This is a complex full-body serial motor skill that is often taught to novices in grappling sports. First, the participant grips the opponent behind their neck and circles their hand to the inside of the opponent's wrist to grab it and gain control of their arm. Then, the participant takes a step back while pulling on the opponent to get the opponent to plant their foot in front of them. Next, the participant lowers themself while pulling on the back of the opponent's neck to break their posture and to force the opponent to shift their weight onto that forward planted foot. Finally, the participant grabs the back of the opponent's ankle of their forward foot and scoops that ankle toward themselves and then drives with their legs to stand back up, whilst guiding their opponent's head back at the same time to complete the takedown and then returning to their starting standing position. This movement was performed with the first author as an opponent for all participants in all testing sessions but physically practiced without an opponent during acquisition sessions (i.e., a shadow movement; e.g., Chye et al., 2024).

### Measures of learning

#### Motor skill performance

**Figure 3:**
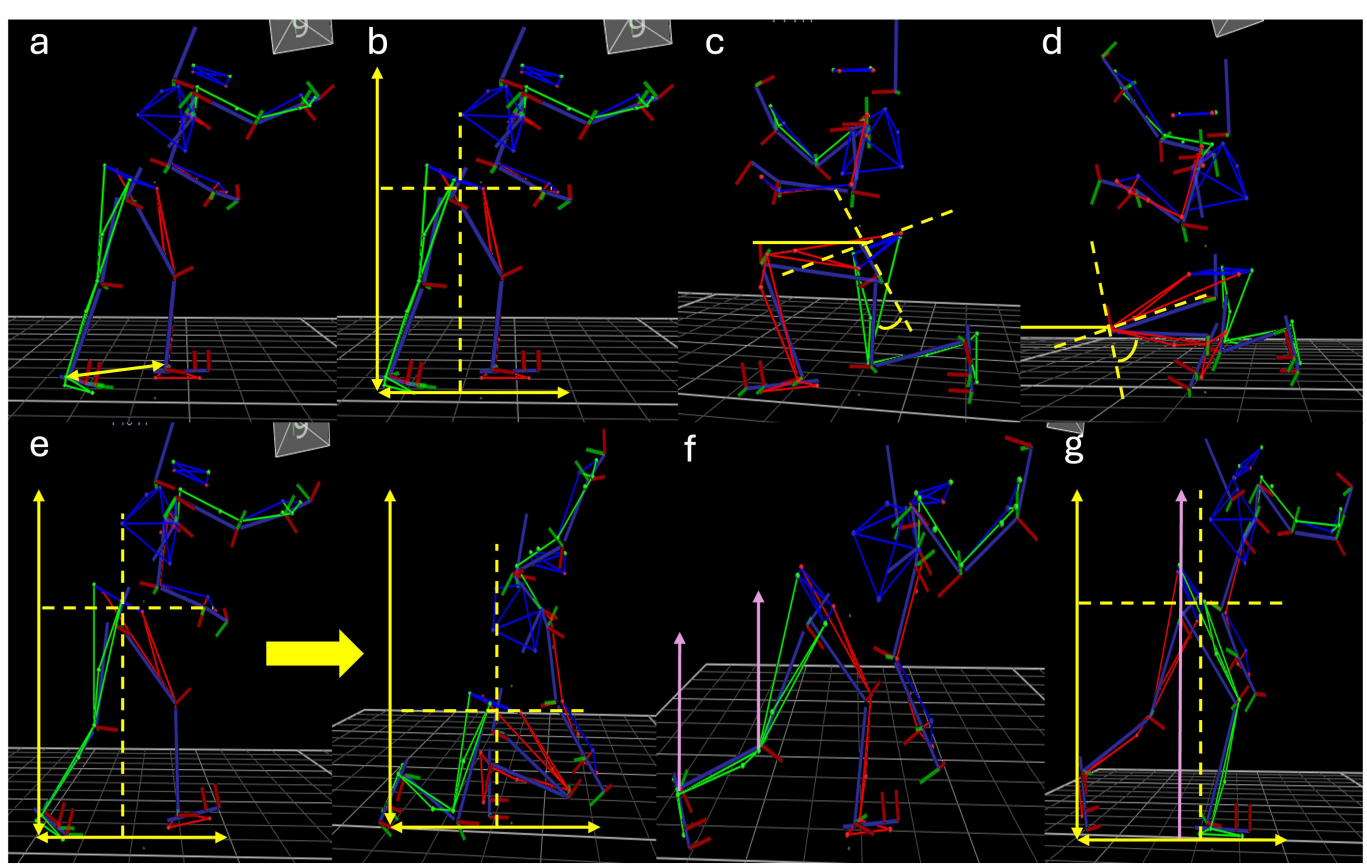
Kinematic models of the data extraction points for biomechanical kinematic markers underpinning successful movements as a measure of motor skill performance in this study. Panels display extraction points for the **(a)** base of support, **(b)** and horizontal and vertical center of mass during the stance phase, **(c)** peak left hip flexion angle, **(d)** peak left knee flexion angle, **(e)** and change in horizontal and vertical center of mass during lowering phase, **(f)** peak right knee peak right ankle extension velocities, and **(g)** peak vertical center of mass velocity during the propulsive phase. Peak angle time difference is calculated based on the time between **(c, d)**, mean vertical velocity is extracted for the lowering phase depicted in the two frames for **(e)**.

Motor skill performance was measured at all three test phases using biomechanical kinematic markers underpinning successful execution of the Ankle Pick takedown (see Figure 3 for a visual depiction of the extracted measures). Ten movement trials were collected at each test phase, with a total of thirty successful movement trials completed per participant across the study period. Three-dimensional marker positions were recorded using a 12 camera Vicon Vantage motion capture system (Vicon, Oxford, UK) sampling at 100 Hz. Participants wore 39 reflective skin markers at selective anatomical landmarks according to the Vicon plug-in gait marker set, which were tracked throughout all movement trials and filtered using a Butterworth fourth order low pass filter with a cut-off frequency of 6 Hz and then used to create a whole-body model. Twelve discrete kinematic variables were extracted from each successful test trial of the Ankle Pick takedown across the three phases of the movement. Three discrete kinematic variables were extracted from the Stance phase of the Ankle Pick takedown: base of Support, Horizontal Center of Mass, and Vertical Center of Mass. Six discrete kinematic variables were extracted from the Lowering phase of the Ankle Pick takedown: peak Left Hip Flexion Angle, Peak Left Knee Flexion Angle, Peak Angle Time Difference, Change in Horizontal Center of Mass, Change in Vertical Center of Mass, and Average Vertical Velocity. Three discrete kinematic variables were extracted from the Propulsive phase of the Ankle Pick takedown: peak Right Knee Extension Velocity, Peak Right Ankle Extension Velocity, and Peak Vertical Velocity (detailed in Table 1). A skilled grappling athlete performed 10 successful trials of the Ankle Pick takedown and the mean score for each kinematic variable was used as a reference value. The absolute error score (i.e., difference between the participant's recorded value and the reference value) was calculated using an average score of the participants 10 movement trials in each test phase, with a lower error score for each discrete kinematic measure representing more successful motor skill performance.

**Table 1:** Discrete kinematic measures recorded to represent motor skill performance for the Ankle Pick takedown.

**Movement phase**	**Discrete kinematic measure**	**Description**	**Interpretation**	**Determination**
Stance	Base of Support (%)	Horizontal displacement between the right and left ankle (normalized to leg length) within the Base of Support at the end of the Stance phase prior to lowering	Provides insight on the stability of the body	Horizontal distance calculated between the right and left ankle marker and reported as a percentage of leg length
	Horizontal Center of Mass (%)	Horizontal position of the Center of Mass within the Base of Support at the end of the Stance phase prior to lowering	Provides insight on the stability of the body	Position of Center of Mass is reported as a percentage of Base of Support
	Vertical Center of Mass (%)	Vertical position of the Center of Mass within the Base of Support at the end of the Stance phase prior to lowering	Provides insight on the height of the Center of Mass from the ground	Position of Center of Mass is reported as a percentage of leg length
Lowering	Peak Left Hip Flexion Angle (degrees)	Minimum/Peak Left Hip Flexion Angle during the lowering phase	Provides insight on the minimum trunk flexion during the downward motion of the center of mass	Hip Flexion Angle is determined from the Vicon Plug-in Gait model from the angle between the torso and upper leg vector using the vector dot product between the two segments
	Peak Left Knee Flexion Angle (degrees)	Minimum/Peak Left Knee Flexion Angle during the lowering phase	Provides insight on the optimum knee angle for generation of force	Knee Flexion Angle is determined from the Vicon Plug-in Gait model from the angle between the lower leg and upper leg vector using the vector dot product between the two segments
	Peak Flexion Angle Time Difference (s)	Time difference between the Minimum/Peak Left Hip and Knee Flexion angle during the lowering phase	Provides insight on the synchronization of the trunk and lower body	Temporal values are calculated from the frame rate of the Vicon system
	Change in Horizontal Center of Mass (m)	Change in the horizontal displacement of the Center of Mass from the initiation of the vertical lowering of the Center of Mass to a minimum	Provides insight on the stability of the body	Position of Center of Mass is determined from the Vicon Plug-in Gait model and reported as a percentage of Base of Support
	Change in Vertical Center of Mass (%)	Change in the vertical displacement of the Center of Mass from the initiation of the vertical lowering of the Center of Mass to a minimum	Provides insight on the height of the Center of Mass from the ground	Position of Center of Mass is determined from the Vicon Plug-in Gait model and reported as a percentage of leg length
	Mean Vertical Velocity (m/s)	Mean vertical velocity of the Center of Mass from the initiation of the vertical lowering of the Center of Mass to a minimum	Provides insight on the control during the lowering phase	Average vertical velocity is calculated from the change in vertical displacement divided by the time of the lowering phase
Propulsive	Peak Right Knee Extension Velocity (deg/s)	Peak Extension Velocity of the right knee during the propulsive phase	Provides insight on the momentum being transferred to upper body and to opponent	Knee extension velocity is determined from the knee angular displacement data (in the sagittal plane) and time using mathematical differentiation
	Peak Right Ankle Extension Velocity (deg/s)	Peak Extension Velocity of the right ankle during the propulsive phase	Provides insight on the momentum being transferred to upper body and to opponent	Ankle extension velocity is determined from the ankle angular displacement data (in the sagittal plane) and time using mathematical differentiation
	Peak Vertical Center of Mass Velocity (m/s)	Peak Vertical Velocity of the Center of Mass during the propulsive phase	Provides insight on the momentum being transferred to upper body and to opponent	Center of Mass velocity is determined from the vertical Center of Mass displacement data and time using mathematical differentiation

#### Self-efficacy

Self-efficacy was assessed using a bespoke 8-item self-report questionnaire developed using efficacy measurement guidelines (Bandura, 2006). The questionnaire was tailored for the Ankle Pick takedown and required participants to make confidence judgments about their ability to successfully perform the different components of the takedown, as well as the overall movement. Participants rated each item on an 11-point Likert scale from 0 (not at all confident) to 10 (completely confident). The specific components of the movement were taken from grappling resources (i.e., wrestling and Brazilian jiu-jitsu tutorials of the takedown) and checked by the first author as a skilled grappling athlete.

#### Mental representation structure

**Figure 4:**
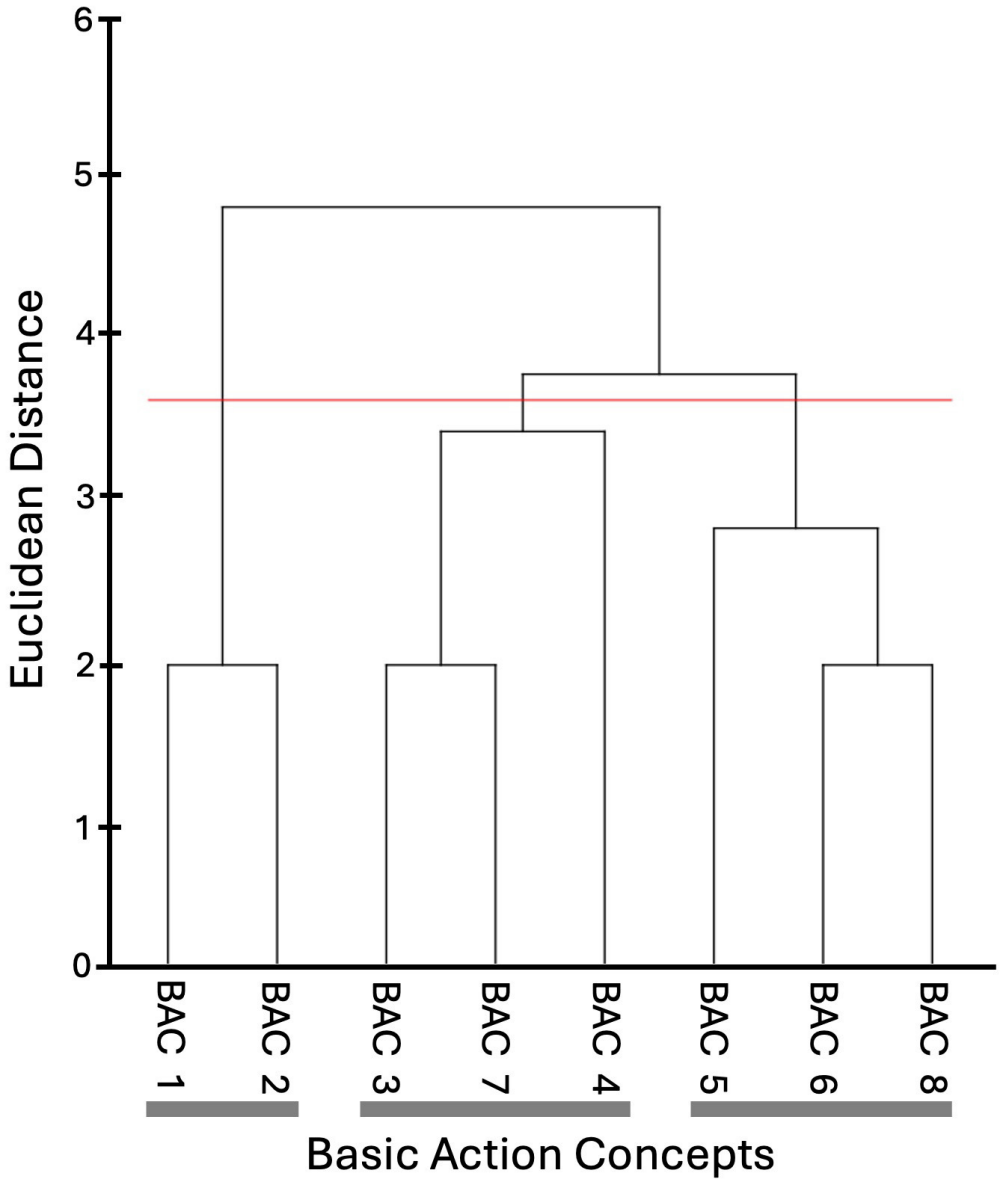
Mean group tree diagram of the Ankle Pick takedown for the reference group of two skilled grappling athletes. Each BAC is labeled on the x-axis (for the list of BACs, see Table 2). The numbers on the y-axis display Euclidean distances. The lower the Euclidean distance between BACs, the closer the BACs are. The horizontal red line marks the critical value *d*_*crit*_ for a given α-level (*d*_*crit*_ = 3.51; α = 0.05). Thick horizontal gray lines below the BAC labels on the x-axis depict clusters of BACs.

Mental representation structure was assessed using structural dimensional analysis of mental representation (SDA-M; Schack, 2012) as an indicator of accurate representation of the Ankle Pick takedown in long-term memory. As per the recommendations of Schack (2012), a list of basic action concepts (BACs) for the Ankle Pick takedown were initially developed by the first author based on her knowledge of the movement and the above-mentioned grappling resources. This list was rated by an independent panel of experts, including three skilled grappling athletes and two coaches with advanced knowledge and experience of performing/teaching the Ankle Pick takedown. A final list of BACs was adapted based on their feedback and used for the SDA-M splitting procedure in this study (Table 2). This involved one BAC being displayed on the screen (the anchor concept) while the participant decided if the other BACs (*n* = 8), which were displayed one after another in a randomized order, were directly related to the anchor concept. Once all decisions were recorded for that anchor concept, the procedure was repeated until all BACs had taken the anchor position, and all decisions had been made. The whole split procedure lasted ~10–20 min for a total of 72 decisions (9 x 8). As two skilled grappling athletes, the first author and a member of the expert panel completed the SDA-M splitting procedure to create a “skilled performer” mental representation structure for the Ankle Pick takedown as a reference point for this study (Figure 4).

**Table 2:** Basic action concepts of the Ankle Pick takedown.

**Number**	**Basic action concept (BAC)**
1	Right hand pulls down on partner's neck
2	Lower body and center of mass
3	Drop left knee toward the ground
4	Contact between left hand and partner's right ankle
5	Left hand scoops up their right ankle
6	Drive forward with body
7	Right hand guides partner's head backwards and downwards
8	Right leg drives movement to stand back up

### Training conditions

#### Self-modeled AOMI (AOMI_*SELF*_)

In the AOMI_SELF_ training condition, participants watched a 4-s video of a skeleton-like avatar representing her/his own successful performance of the Ankle Pick takedown and simultaneously imagined the kinesthetic sensations involved with performing the movement on an opponent. The skeleton model was chosen based on evidence suggesting that anthropomorphic-like displays can effectively convey kinematic information and enhance perception of motion features compared to simpler representations, such as point-light displays or stick figures (e.g., Hodgins et al., 1998; Moura et al., 2023). The movements of the skeleton-like avatar were shown in both egocentric (Figure 2b) and allocentric visual perspectives (Figure 2c) in an alternating fashion (Chye et al., 2024). A three-dimensional model of the participant's most successful trial of the Ankle Pick takedown at pre-test was imported from Vicon Nexus into Vicon Polygon to create the skeleton-like avatar video (see Supplementary material for a detailed account of Vicon Polygon processes). The most successful three trials were determined based on having the three lowest error scores across the twelve kinematic measures compared to the skilled model's kinematic data. These trials were subjectively rated by the first author, as a skilled grappling athlete, to determine the objectively and subjectively most successful trial used for the skeleton-like avatar in the AOMI_SELF_ training condition. All test trials were performed with an opponent, but only the movement of the participant was motion-captured and thus represented as the skeleton-like avatar in the videos. Whilst watching the videos, the participants were instructed to perform their imagery as though they were throwing an opponent, focusing on the physical sensations of the movement based on their kinesthetic experience of it from the test trials. During each training session, the participant engaged with sixty AOMI_SELF_ trials broken down into six blocks of ten trials, totalling 240 s. After 5 consecutive AOMI_SELF_ trials, the participant performed 5 physical practice trials of the Ankle Pick takedown without an opponent, meaning a total of 60 physical practice trials were completed per training session. Physical practice was incorporated as part of the AOMI training interventions in this study as novices benefit most from pairing movement simulation and execution due to limited experience performing the skill (McNeill et al., 2020).

#### Other-modeled AOMI (AOMI_*OTHER*_)

The AOMI_OTHER_ training condition was identical to the AOMI_SELF_ training condition, with the only difference being the content displayed in the skeleton-like avatar video trials. The skeleton-like avatar represented the three-dimensional model from the motion capture of a skilled athlete performing the Ankle Pick takedown successfully on an opponent, but only the movement of the skilled model was represented as the skeleton-like avatar in the videos, as shown in both egocentric (Figure 2d) and allocentric visual perspectives (Figure 2e) in an alternating fashion (Chye et al., 2024). During each training session, the participant engaged with sixty AOMI_OTHER_ trials broken down into six blocks of ten trials, totalling 240 s. After 5 consecutive AOMI_OTHER_ trials, the participant performed 5 physical practice trials of the Ankle Pick takedown without an opponent, meaning a total of 60 physical practice trials were completed per training session.

#### Control

Participants allocated to the Control training condition watched video extracts from an interview with a professional grappling athlete, a common control condition used in the AOMI literature (see Figure 1 for an image depicting this). The videos did not include any technical information about the Ankle Pick takedown and focused on the athlete's general experiences as a competitor. During each training session, the participant watched six 40-s extracts from the recorded interview to match the total duration for 60 repetitions of the intervention footage used in the AOMI_SELF_ and AOMI_OTHER_ training conditions. After watching each 40-s extract, the participant performed 10 physical practice trial of the Ankle Pick takedown, meaning a total of 60 physical practice trials were completed per training session.

### Procedure

#### Familiarization and pre-test phase

The participant arrived at the biomechanics laboratory to complete the pre-test data collection (Day 1). Participants were initially briefed on the study requirements and provided informed consent, before being prepared for motion capture data collection. Participants were then shown a demonstration video that displayed a skilled grappling athlete performing four Ankle Pick takedowns on an “opponent.” The first two repetitions demonstrated the movement in chunks with stepwise verbal instructions and the second two repetitions demonstrated the takedown at full speed without instruction. Participants then practiced five repetitions of the takedown without an “opponent,” and then five more repetitions of the takedown with an “opponent.” Feedback of the performance was given if there were any fundamental steps missing or were performed incorrectly. Participants then completed the self-efficacy questionnaire and SDA-M procedure. To complete the pre-test phase, participants then performed ten “test” repetitions of the Ankle Pick takedown on an “opponent” whilst kinematic data was collected.

#### Acquisition phase

All participants took part in 5 training sessions during the acquisition phase of the experiment (Days 3–7). For each session, participants were first led through a 5-min dynamic warm up that included all muscle groups used during the Ankle Pick takedown. Participants then watched the demonstration video to re-familiarize with the different components of the takedown when performed on another person, then watched another demonstration video of the takedown performed without an ‘opponent' to demonstrate what the takedown would look like during their physical practice trials without an “opponent.” Participants then received instructions based on their allocated training condition (see Supplementary files for full instructions for each training condition) and engaged in the training protocol. For all training conditions, the participant completed a total of 13.5-min non-physical practice (i.e., engagement with the stimuli and task associated with their allocated training condition) and 300 physical practice trials without an “opponent” for the Ankle Pick takedown.

#### Post-test and retention-test phases

Participants returned to the biomechanics laboratory 1 day and 8 days after finishing the final training session of the acquisition phase to complete the post-test (Day 8) and retention-test (Day 15) data collections, respectively. A buffer day was included between the pre-test (Day 1) and the start of the acquisition phase (Day 3) to allow time for processing data and preparing the materials for the AOMI_SELF_ condition from the data collected in the pre-test session. The protocol for both data collection sessions mirrored that of the pre-test, recording movement kinematics, self-efficacy, and mental representation structures for the Ankle Pick takedown for a second and third instance across the study period. At the end of the experiment, participants allocated to the AOMI_SELF_ and AOMI_OTHER_ training conditions completed a social validation questionnaire and interview. The social validation procedures aimed to gather perceived changes in motor skill performance, self-efficacy and mental representations for the training condition, and assess perceived ability to engage with the simulation processes and embody the movements displayed by the avatar during the AOMI training protocol (see Supplementary material for full details and reporting of social validation data).

### Kinematic data extraction

The twelve discrete kinematic variables were extracted from significant phases during the ankle pick movement. Their descriptions, interpretations and methods of determination, can be found in full in Table 2.

### Data analysis

#### Motor skill performance and self-efficacy

Multi-level linear models (MLM) were run for motor skill performance and self-efficacy data using the “lme4” package in R studio statistical software (version 4.2.1). Significance was calculated using the “lmerTest” package (Kuznetsova et al., 2017), which applies Satterthwaite's method to estimate degrees of freedom and generate *p*-values for mixed models. Mean overall self-efficacy scores and absolute error scores for each discrete kinematic measure served as separate dependent variables, and “participant” was included as a random intercept. We attempted to model random slopes to account for individual differences in response across test phases, but the model would not run because it lacked sufficient data to reliably estimate all the specified random effects parameters. Outlier analyses were computed for motor skill performance and self-efficacy data using interquartile range values. Forty-two individual data points were removed as outliers across the thirteen MLM.

To check for any potential effects of imagery ability on the respective scores, a second identical model was run with kinesthetic imagery scores from the VMIQ-2 (Roberts et al., 2008) added as a covariate for each of the dependent variables. The MLMs incorporating imagery ability as a co-variate increased the accuracy of the models compared to the original models for three of the thirteen outcome measures (i.e., Peak Flexion Angle Time Difference, Peak Right Knee Extension Velocity, and Peak Right Ankle Extension Velocity), meaning these secondary models are reported in the main results below. Model accuracy and influence statistics for the original or secondary MLMs not reported in the primary results section are reported in the Supplementary Results document. Random effect residuals for “participant” accounted for a significant portion of the variance across all kinematic variables as well as self-efficacy scores, supporting the decision to model these random effects in the MLMs (see Supplementary Table S1 in the Supplementary Results document for additional detail).

To account for potential washout effects in the motor skill performance data that may have resulted from the large trial-count used across the testing procedures, we loaded “trial count” as a factor into the MLMs as a continuous fixed effect. This was calculated using a sequential moving average (i.e., trial count n is represented by the average of trials 1-n) and the outliers removed from the main analysis were also excluded from this analysis. We only considered the data at post-test and retention-test as the pre-test data was taken prior to the intervention, meaning washout is not an issue at this point. There was no main effect of trial count for most kinematic variables at post-test (*n* = 9) and retention-test (*n* = 7). An interaction effect of trial count x training condition was present for two kinematic variables at post-test and at retention-test. Follow-up pairwise comparisons for Horizontal Center of Mass showed that error scores for the AOMI_OTHER_ training condition were significantly lower at trial 1 compared to trials 3–10 at post-test. Follow-up pairwise comparisons for Peak Flexion Angle Time Difference showed no significant differences in error scores for the AOMI_OTHER_ training condition across trial counts at post-test. Follow-up pairwise comparisons for Change in Horizontal Center of Mass showed that error scores for the AOMI_OTHER_ training condition were significantly lower at trial 1 compared to trials 2 and 3 at retention-test. Follow-up pairwise comparisons for Peak Right Ankle Extension Velocity showed that error scores for the AOMI_SELF_ training condition were significantly higher at trial 1 compared to trials 3–10, and at trial 2 compared to trials 9 and 10 at retention-test. These analyses suggest there is no systematic presence of a washout effect due to the number of trials used for the testing procedures adopted in this study.

#### Mental representation structure

Drawing on the Euclidean distance scaling between BACs as obtained by the SDA-M split procedure, cluster analyses (α = 0.5, *d*_*crit*_ =3.51) were performed to outline the structure of mental representations. Mean group tree diagrams were computed for each experimental condition (AOMI_SELF_, AOMI_OTHER_, Control) at each test phase (Pre-Test, Post-Test, and Retention-Test). Analysis of invariance was conducted to compare the different cluster solutions between training conditions and across test phases. Two cluster solutions are variant when λ < 0.68 and are invariant when cluster solutions are λ ≥ 0.68 (Schack, 2012). Closer proximity with the reference structures indicates a more functionally accurate representation of the Ankle Pick takedown. The Adjusted Rand Index (ARI; Santos and Embrechts, 2009) was calculated as a similarity metric between the structures for the training conditions and the reference structure at each test phase. ARI values between −1 (structures are different) and 1 (structures are the same) were obtained, with a greater positive difference in ARI values between pre-test and retention-test indicating greater learning of the cognitive aspects underlying physical execution of the Ankle Pick takedown.

## Results

### Motor skill performance

#### Stance phase

**Figure 5:**
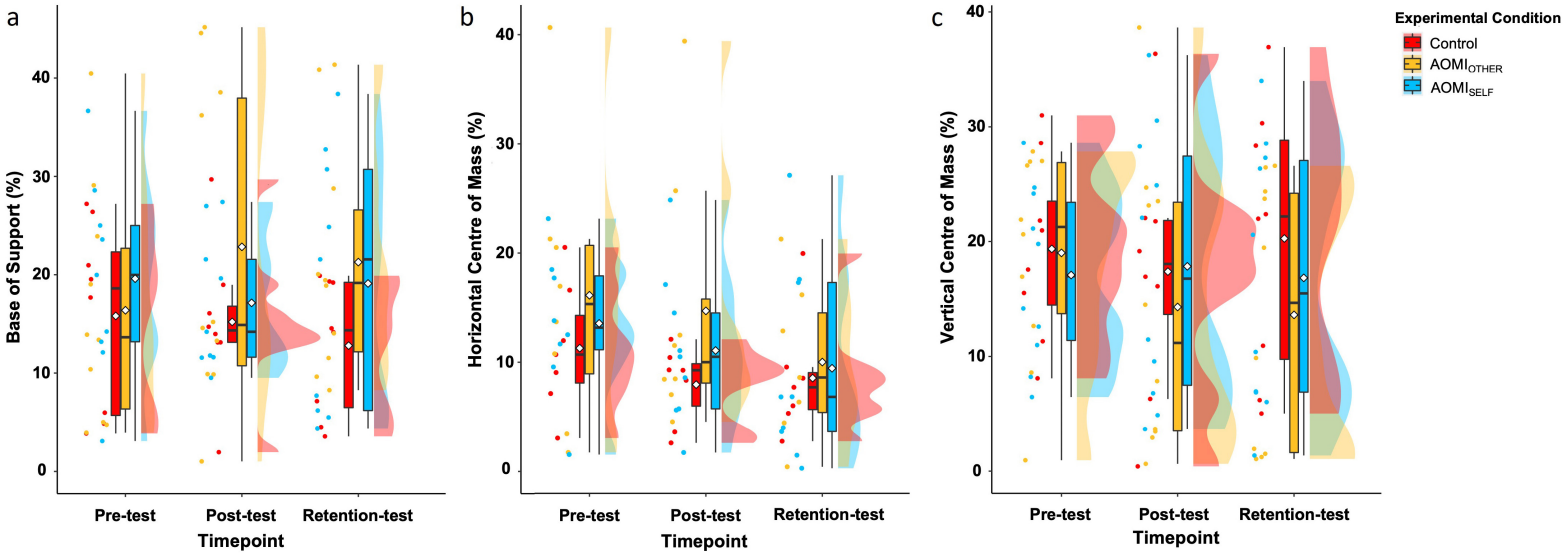
Box and violin plots with raw data points displaying error scores for **(a)** Base of Support, **(b)** Horizontal Center of Mass, and **(c)** Vertical Center of Mass discrete kinematic measures for the stance phase of the Ankle Pick Takedown in the Control (red), AOMI_OTHER_ (yellow), and AOMI_SELF_ (blue) training conditions across the three test phases. Thick horizontal black lines represent the median average and white diamonds represent the mean average for each box plot. Individual participant error scores are represented by circular markers. Data points are omitted for those excluded through outlier diagnostics.

**Base of support**. There was no significant main effect of test phase (*F* [2, 53.47] = 0.06, *p* = 0.94, η^2^ = 0.002), training condition (*F* [2, 27.28] = 1.08, *p* = 0.35, η^2^ = 0.07) or interaction effect between training condition and test phase (*F* [4, 53.46] = 1.16, *p* = 0.34, η^2^ = 0.08) for Base of Support error scores (Figure 5a).

**Horizontal center of mass**. There was a significant main effect of test phase (*F* [2, 44. 8] = 3.5, *p* = 0.04, η^2^ = 0.13), but no significant main effect of training condition (*F* [2, 24.15] = 1, *p* = 0.41, η^2^ = 0.07) or interaction effect between training condition and test phase (*F* [4, 44.8] = 0.21, *p* = 0.93, η^2^ = 0.02) for Horizontal Center of Mass error scores (Figure 5b). Follow-up pairwise comparisons for the main effect of test phase suggested that Horizontal Center of Mass error scores did not significantly differ between pre-test and post-test (ß = 2.24, *t*_(52.7)_ = 1.73, *p* = 0.2), between pre-test and retention- test (ß = 3.14, *t*_(52.9)_ = 2.4, *p* = 0.052), and between post-test and retention test (ß = 0.91, *t*_(52)_ = 0.71, *p* = 0.76).

**Vertical center of mass**. There was no significant main effect of test phase (*F* [2, 56] = 1.04, *p* =.36, η^2^ = 0.04), training condition (*F* [2, 28] = 0.3, *p* = 0.74, η^2^ = 0.02), or interaction effect between training condition and test phase (*F* [4, 56] = 1.27, *p* = 0.29, η^2^ = 0.08) for Vertical Center of Mass error scores (Figure 5c).

#### Lowering phase

**Figure 6:**
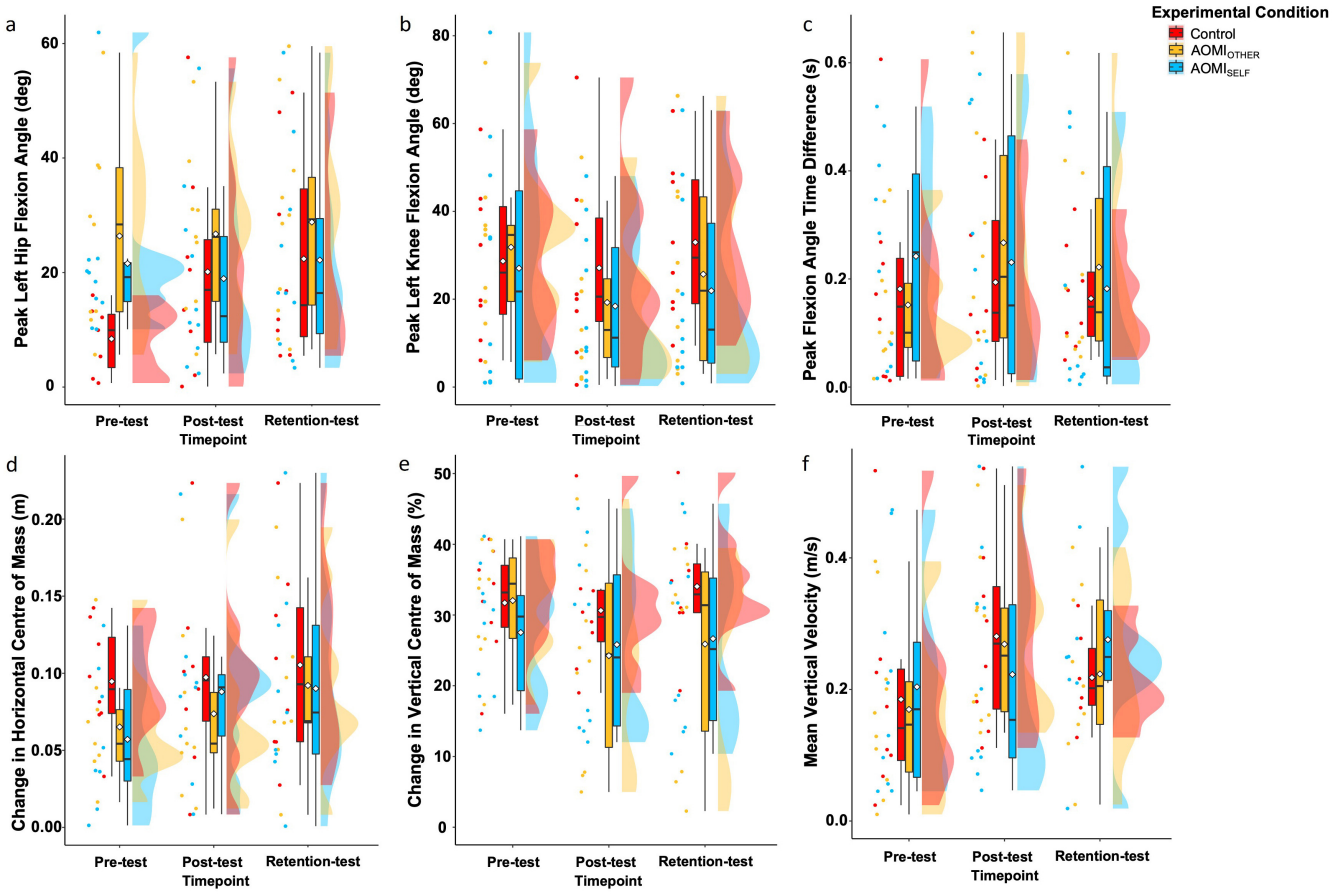
Box and violin plots with raw data points displaying error scores for **(a)** Peak Left Hip Flexion Angle, **(b)** Peak Left Knee Flexion Angle, **(c)** Peak Flexion Angle Time Difference, **(d)** Change in Horizontal Center of Mass, **(e)** Change in Vertical Center of Mass, and **(f)** Mean Vertical Velocity discrete kinematic measures for the lowering phase of the Ankle Pick takedown in the Control (red), AOMI_OTHER_ (yellow), and AOMI_SELF_ (blue) training conditions across the three test phases. Thick horizontal black lines represent the median average and white diamonds represent the mean average for each box plot. Individual participant error scores are represented by circular markers. Data points are omitted for those excluded through outlier diagnostics.

**Peak left hip flexion angle**. There was no significant main effect of test phase (*F* [2, 50.4] = 0.83, *p* = 0.44, η^2^ = 0.03), training condition (*F* [2, 24.42] = 1.79, *p* = 0.19, η^2^ = 0.13), or interaction effect between training condition and test phase (*F* [4, 50.37] = 0.66, *p* = 0.62, η^2^ = 0.05) for Peak Left Hip Flexion Angle error scores (Figure 6a).

**Peak left knee flexion angle**. There was no significant main effect of test phase (*F* [2, 54] = 2.03, *p* = 0.14, η^2^ = 0.07), training condition (*F* [2, 27) = 0.43, *p* = 0.65, η^2^ = 0.03). or interaction effect between training condition and test phase (*F* [4, 54] = 0.47, *p* = 0.76, η^2^ = 0.03) for Peak Left Knee Flexion Angle error scores (Figure 6b).

**Peak flexion angle time difference**. There was no significant main effect of test phase (*F* [2, 56] = 0.87, *p* = 0.42, η^2^ = 0.03), training condition (*F* [2, 28] = 0.17, *p* = 0.84, η^2^ = 0.01) or interaction effect between training condition and test phase (*F* [4, 56] = 0.87, *p* = 0.49, η^2^ = 0.06) for Peak Flexion Angle Time Difference error scores (Figure 6c).

**Change in horizontal center of mass**. There was no significant main effect of test phase (*F* [2, 48.83] = 1.44, *p* = 0.25, η^2^ = 0.06), training condition (*F* [2, 24] = 0.81, *p* = 0.46, η^2^ = 0.06) or interaction effect between training condition and test phase (*F* [4, 48.8] = 0.2, *p* = 0.94, η^2^ = 0.02) for Change in Horizontal Center of Mass error scores (Figure 6d).

**Change in vertical center of mass**. There was no significant main effect of test phase (*F* [2, 56] = 2.41, *p* = 0.1, η^2^ = 0.08), training condition (*F* [2, 28] = 0.83, *p* =.45, η^2^ = 0.06) or interaction effect between training condition and test phase (*F* [4, 56] = 1.45*, p* = 0.23, η^2^ = 0.09) for Change in Vertical Center of Mass error scores (Figure 6e).

**Mean Vertical Velocity**. There was no significant main effect of test phase (*F* [2, 53.8] = 2.42, *p* = 0.1, η^2^ = 0.08), training condition (*F* [2, 28.53] = 0.07, *p* = 0.93, η^2^ = 0.005) or interaction effect between training condition and test phase (*F* [4, 53.74] = 0.6, *p* = 0.67, η^2^ = 0.04) for Mean Vertical Velocity error scores (Figure 6f).

#### Propulsive phase

**Figure 7:**
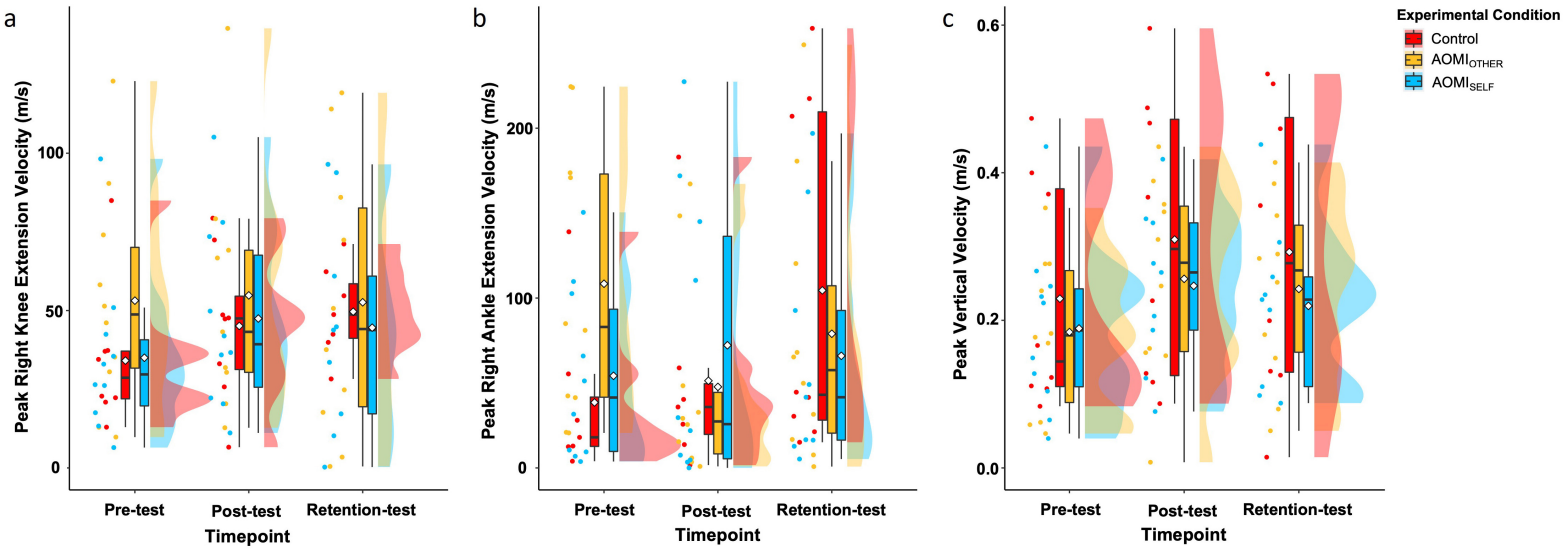
Box and violin plots with raw data points displaying error scores for **(a)** Peak Right Knee Extension Velocity, **(b)** Peak Right Ankle Extension Velocity, and **(c)** Peak Vertical Velocity discrete kinematic measures for the propulsive phase of the Ankle Pick takedown in the Control (red), AOMI_OTHER_ (yellow), and AOMI_SELF_ (blue) training conditions across the three test phases. Thick horizontal black lines represent the median average and white diamonds represent the mean average for each box plot. Individual participant error scores are represented by circular markers. Data points are omitted for those excluded through outlier diagnostics.

**Peak right knee extension velocity**. There was no significant main effect of test phase (*F* [2, 53.77] = 1.11, *p* = 0.34, η^2^ = 0.04), training condition (*F* [2, 27.93] = 0.58, *p* = 0.56, η^2^ = 0.04), or interaction effect between training condition and test phase (*F* [4, 53.75] = 0.38, *p* = 0.82, η^2^ = 0.03) for Peak Right Knee Extension Velocity error scores (Figure 7a).

**Peak right ankle extension velocity**. There was no significant main effect of test phase (*F* [2, 51.69] = 1.3, *p* = 0.28, η^2^ = 0.05), training condition (*F* [2, 25.63] = 0.15, *p* = 0.86, η^2^ = 0.01), or interaction effect between training condition and test phase (*F* [4, 51.63] = 2.22, *p* = 0.08, η^2^ = 0.15) for Peak Right Ankle Angle Velocity error scores (Figure 7b).

**Peak vertical velocity**. There was a significant main effect of test phase (*F* [2, 53.41] = 7.41, *p* < 0.01, η^2^ = 0.22), but no significant main effect of training condition (*F* [2, 27.03] = 0.46, *p* =.64, η^2^ = 0.03) or interaction effect between training condition and test phase (*F* [4, 53.41] = 0.03, *p* = 1, η^2^ = 0.002) for Peak Vertical Velocity error scores (Figure 7c). Follow-up pairwise comparisons for the main effect of test phase suggested that Peak Vertical Velocity error scores significantly increased between pre-test and post-test (= −0.08, *t*_(61.3)_ = −3.49, *p* < 0.01) and between pre-test and retention-test (ß = −0.06, *t*_(61.3)_ = −2.61, *p* = 0.03), but did not differ significantly between post-test and retention-test (ß = 0.02, *t*_(60.8)_ = 0.88, *p* = 0.66).

#### Self-efficacy

**Figure 8:**
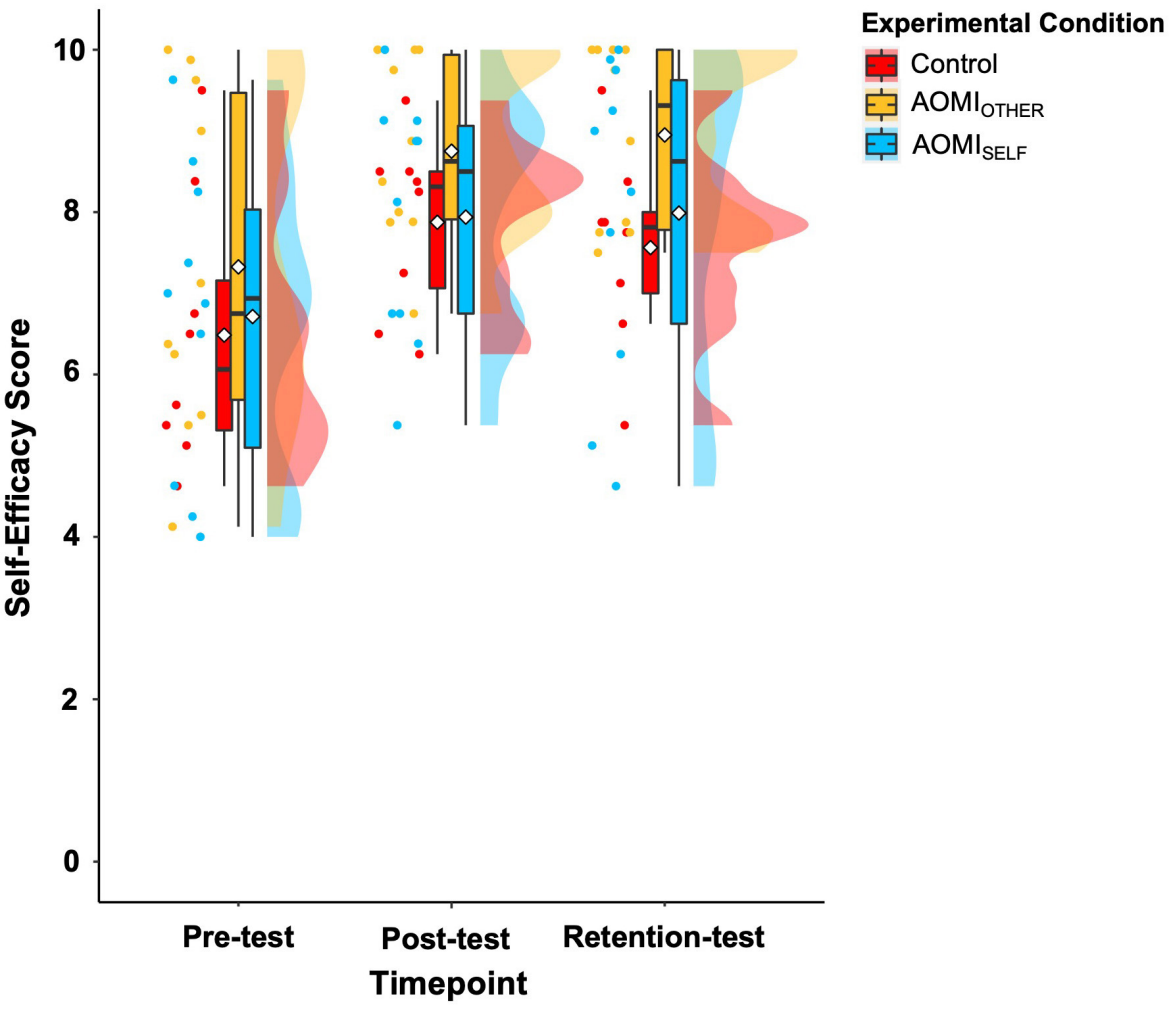
Box and violin plots with raw data points displaying self-efficacy scores in the Control (red), AOMI_OTHER_ (yellow), and AOMI_SELF_ (blue) training conditions across the three test phases. Thick horizontal black lines represent the median average and white diamonds represent the mean average for each box plot. Individual participant error scores are represented by circular markers. Data points are omitted for those excluded through outlier diagnostics.

There was a significant main effect of test phase (*F* [2, 56] = 27.06, *p* < 0.001, η^2^ = 0.49), no significant main effect of training condition (*F* [2, 28] = 1.45, *p* = 0.25, η^2^ = 0.09) or interaction effect between training condition and test phase (*F* [4, 56] = 0.39, *p* = 0.82, η^2^ = 0.03) for self-efficacy scores (Figure 8). Follow-up pairwise comparisons for the main effect of test phase suggested that self-efficacy scores significantly increased between pre-test and post-test (ß = −1.35, *t*_(62.7)_ = −6.07, *p* < 0.001) and between pre-test and retention-test (ß = −1.33, *t*_(62.7)_ = −5.92, *p* < 0.001) but did not significantly differ between post-test and retention test (ß = 0.02, *t*_(62.7)_ = 0.1, *p* = 1).

### Mental representation structure

#### Self-model AOMI (AOMI_*SELF*_) training condition

**Figure 9:**
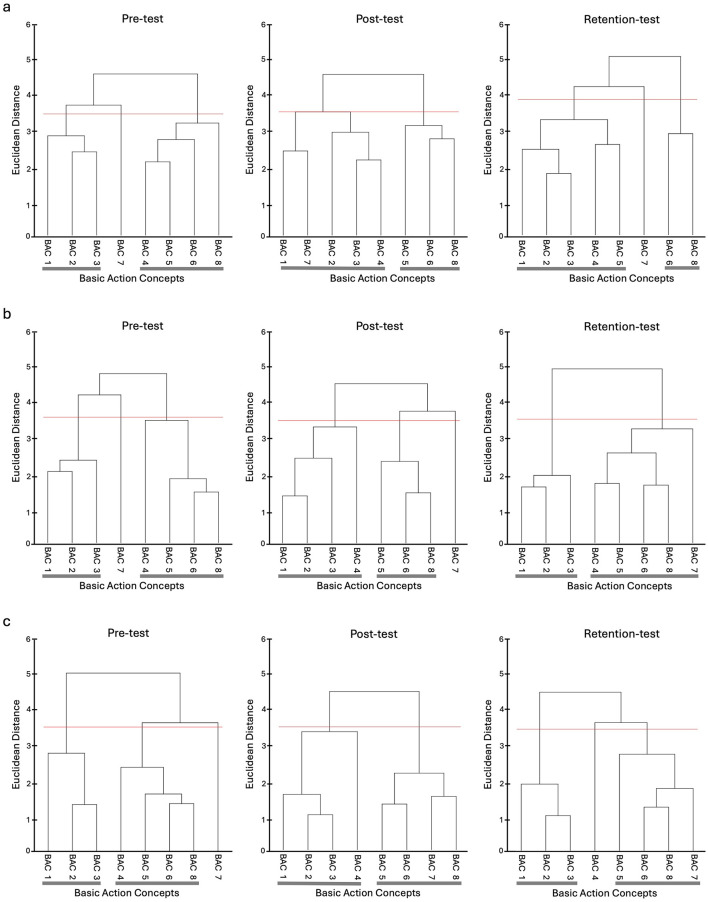
Mean group tree diagram of the Ankle Pick for the **(a)** AOMI_SELF_, **(b)** AOMI_OTHER_, and **(c)** Control training conditions across the three test phases. Each BAC is labeled on the x-axis (for the list of BACs, see Table 2). The numbers on the y-axis display Euclidean distances. The lower the Euclidean distance between BACs, the closer the BACs are. The horizontal red line marks the critical value *d*_*crit*_ for a given α-level (*d*_*crit*_ = 3.51; α = 0.05). Thick horizontal gray lines below the BAC labels on the x-axis depict clusters of BACs.

The mean group tree diagrams (Figure 9a) for participants in the AOMI_SELF_ training condition comprised of two clusters at pre-test (BACS [1 2 3]; [4 5 6 8]), two clusters at post-test (BACS [1 7 2 3 4]; [5 6 8]) and two clusters at retention-test (BACS [1 2 3 4 5]; [6 8]). Analysis of invariance revealed that the representation structures for participants allocated to the AOMI_SELF_ training condition were invariant between pre-test and post-test (λ = 0.69), pre-test and retention-test (λ = 0.72) and post-test and retention-test (λ = 0.7). Mental representation structures became less like the model structure over time between pre-test and post-test (*ARI*_*pre*__=_ 0.13, *ARI*_*post*_ = −0.04, *ARI*_*diff*_ = −0.17), pre-test and retention-test (*ARI*_*pre*__=_ 0.13, *ARI*_*retention*_ = −0.12*, ARI*_*diff*_ = –0.25), and post-test and retention-test (*ARI*_*post*_ = −0.04, *ARI*_*retention*_ = −0.12, *ARI*_*diff*_ = −0.08).

#### Other-model AOMI (AOMI_*OTHER*_) training condition

The mean group tree diagrams (Figure 9b) for participants in the AOMI_OTHER_ training condition comprised of two clusters at pre-test (BACS [1 2 3]; [4 5 6 8]), two clusters at post-test (BACS [1 2 3]; [5 6 8]) and two clusters at retention-test (BACS [1 2 3]; [4 5 6 8 7]). Analysis of invariance revealed that the representation structures for participants allocated to the AOMI_OTHER_ condition were invariant between pre-test and post-test (λ = 0.71), pre-test and retention-test (λ = 0.69) and post-test and retention-test (λ = 0.69). Mental representation structures did not get closer to the model structure between pre-test and post-test (*ARI*_*pre*_ = 0.13, *ARI*_*post*_ = 0.13, *ARI*_*diff*_ = 0.00), but became more like the model structure over time between pre-test and retention-test (*ARI*_*pre*_ = 0.13, *ARI*_*retention*_ = 0.26, *ARI*_*diff*_ = +0.13), and post-test and retention-test (*ARI*_*post*_ = 0.13, *ARI*_*retention*_ = 0.26, *ARI*_*diff*_ = +0.13).

### Control training condition

The mean group tree diagrams (Figure 9c) for participants in the Control training condition comprised of two clusters at pre-test (BACS [1 2 3]; [4 5 6 8]), two clusters at post-test (BACS [1 2 3 4]; [5 6 7 8], and two clusters at retention-test (BACS [1 2 3]; [5 6 8 7]). Analysis of invariance revealed that the representation structures for participants allocated to the control condition were invariant between pre-test and post-test (λ = 0.68) and post-test and retention-test (λ = 0.68) but were variant between pre-test and retention-test (λ = 0.66). Mental representation structures got closer to the model between pre-test and post-test (*ARI*_*pre*_ = 0.13, *ARI*_*post*_ = 0.31, *ARI*_*diff*_ = +0.18) and pre-test and retention-test (*ARI*_*pre*_ = 0.13, *ARI*_*retention*_ = 0.30, *ARI*_*diff*_ = +0.17), but became less like the model between post-test and retention-test (*ARI*_*post*_ = 0.31, *ARI*_*retention*_ = 0.30, *ARI*_*diff*_ = −0.01).

### Social validation

Participants allocated to the two AOMI groups responded positively to the social validation questionnaire (see Supplementary Table S2 in the Supplementary Results for full breakdown) in terms of the impact of the different AOMI training conditions on the learning measures (AOMI_SELF_ *M* = 3.17, AOMI_OTHER_ *M* = 2.83; range = −5 to 5), their ability to engage with different aspects of AOMI training (AOMI_SELF_ *M* = 1.00, AOMI_OTHER_ *M* = 0.90; range = −3 to 3), and their perceptions of the skeleton-like avatar displayed during the intervention (AOMI_SELF_ *M* = 1.7, AOMI_OTHER_ *M* = 1.23; range = −3 to 3). There were no significant differences between the two training conditions across the social validation questionnaire responses.

All ten participants (100%) allocated to the AOMI_SELF_ and AOMI_OTHER_ training groups believed their motor skill performance improved after training. The participants that perceived AOMI_OTHER_ training as performance-enhancing suggested that the AOMI stimuli provided useful visual information and technical detail about performing the Ankle Pick takedown. Nine participants (90%) believed their self-efficacy increased after AOMI_OTHER_ training, and all ten participants (100%) believed their self-efficacy increased after AOMI_SELF_ training. The participants that perceived AOMI_OTHER_ training to have a positive effect on their self-efficacy suggested the skeleton-like avatar videos provided them with positive mastery experiences. Finally, nine participants (90%) believed their understanding of the movement improved after AOMI_OTHER_ training and all ten participants (100%) believed their understanding of the movement improved after AOMI_SELF_ training. The participants that perceived AOMI_OTHER_ training to advance their knowledge about the Ankle Pick takedown suggested it provided useful cues such as spatial information that helped them to better understand the movement requirements (see Supplementary Results for full breakdown and representative quotes).

## Discussion

This study investigated the influence of model type on the effectiveness of AOMI training for novices learning an Ankle Pick takedown by comparing the effects of AOMI_OTHER_ and AOMI_SELF_ training on motor skill performance, self-efficacy and mental representation structures. This was done by presenting either the novice's (AOMI_SELF_) or skilled performer's (AOMI_OTHER_) movements whilst controlling the visual characteristics by presenting the same skeleton in the intervention (see e.g., Frank et al., 2023). It was hypothesized that AOMI_OTHER_ training would improve motor skill performance and mental representation structure to a greater extent than AOMI_SELF_ training, and AOMI_SELF_ training would increase novice learners' self-efficacy beliefs to a greater extent than AOMI_OTHER_ training. The results of this study partly supported these predictions. There were no significant improvements in motor skill performance between pre- and post-test or pre- and retention-test for any of the three training conditions. However, descriptively, the AOMI training conditions reduced error scores (AOMI_SELF_ = 50% and AOMI_OTHER_ = 50%) across more discrete kinematic measures of motor skill performance than the Control training condition (25%) between pre- and retention-test. Self-efficacy scores increased across the three test phases for all training conditions, with AOMI_SELF_ causing the descriptively largest increase between pre- and retention-test. Mental representation structures became more functional after Control and AOMI_OTHER_ training, and less functional after AOMI_SELF_ training.

Our results do not provide support for the hypothesis that AOMI training would benefit motor skill performance to a greater extent than Control training, irrespective of the model type used. In fact, we show no significant differences between the training conditions in error score changes across all twelve kinematic measures of motor skill performance. The results of this study build on recent findings that AOMI_SELF_ and AOMI_OTHER_ training have similar benefits toward skilled golfers' performance on a putting task (McNeill et al., 2021), and novice exercisers' learning of a whole-body squat movement (Frank et al., 2023). However, the lack of motor skill performance benefits reported in this study contradict recent meta-analytic findings showing that AOMI training incorporating physical practice has a medium-to-large positive effect on performance of different types of motor skills compared to physical practice alone (Chye et al., 2022). This is particularly surprising for AOMI_OTHER_ training given the prevalence of this approach across the studies synthesized in Chye et al.'s (2022) meta-analysis, and the diversity of movement outcome benefits reported for this type of AOMI training to-date. The findings may be in-part explained by the difficulty level of the motor skill being learned in this study, as it is notably more complex than most motor skills employed across previous AOMI training studies with novice populations. A recent study by Chye et al. (2024) found that AOMI training improved some components of motor skill performance at retention-test for novices learning a shadow judo throw that is similar in terms of motor skill classification and movement difficulty to the grappling takedown used in this study. However, a shadow Osoto Gari throw was used in both training and testing in the Chye et al. (2024) study, whereas the Ankle Pick takedown used in this study was trained as a shadow movement but performed on an opponent in the testing sessions, potentially increasing the complexity of the skill and the cognitive processing demands required of the novice learners during test trials in this study. Descriptively, both AOMI training conditions showed reduced error scores across a greater proportion (*n* = 6, 50%) of the twelve kinematic measures of motor skill performance compared to the Control training condition (*n* = 3, 25%) at retention-test, potentially indicating that motor skill performance and learning might be significantly improved if a longer training period was adopted (e.g., Romano-Smith et al., 2018, 2019; Scott et al., 2023), or if the sample size was increased given the small sample size in the present study (Lohse et al., 2016; Ranganathan et al., 2022).

Our results provide partial support for the prediction that AOMI_SELF_ training would lead to the greatest benefits for self-efficacy scores due to model similarity reinforcing positive mastery experiences (Bandura, 1997; Wright et al., 2022). All training conditions increased their self-efficacy scores over time with no significant differences in change scores between the training conditions, but descriptively the AOMI_SELF_ training condition had the largest increase in self-efficacy between pre- and retention-test. This descriptive benefit is likely due to the novice learner seeing successful execution of the movement based on their own motor repertoire in the AOMI_SELF_ training, making successful performance of the task feel more attainable (Shearer et al., 2020; Wright et al., 2022). This is supported by the social validation data collected in this study, as the participants perceived that AOMI_SELF_ training increased self-efficacy through the provision of positive mastery experiences (see Supplementary Results section for more details). The self-efficacy results for this study mirror the findings reported across AO literature in sport, where both self- and other-modeling have been shown to enhance self-efficacy (e.g., Feltz et al., 1979, 2008; Singleton and Feltz, 1999) as well as having no such benefit toward self-efficacy beliefs compared to control conditions (e.g., Law and Ste-Marie, 2005; Ram and McCullagh, 2003; Ste-Marie et al., 2011). Past performance accomplishments are the strongest antecedent for self-efficacy as they provide direct mastery information (Bandura, 1997; Bruton et al., 2013). In the context of this study, the lack of significant differences in self-efficacy scores between training conditions indicates that the novice learners predominantly derived their self-efficacy beliefs from the physical practice of the Ankle Pick takedown, as incorporated for all three groups, possibly due to a lack of direct mastery experiences for this complex motor task.

Our results provide partial support for the prediction that the AOMI_OTHER_ training condition would lead to a more functional mental representation structure for the Ankle Pick takedown in novice learners. Whilst the Control training condition had the largest significant functional change between pre- and retention-test, the AOMI_OTHER_ training condition was the only group that became more like the reference structure between both pre- and post-test and post- and retention-test, indicating a delayed consolidation of the novices' understanding of the movement for this training condition. The lack of significant findings for the AOMI training groups does not align with previous research showing that action simulation training (i.e., AO, MI, AOMI) that incorporates physical practice leads to greater functional changes in novices' mental representation structures of a motor skill compared to physical practice alone (e.g., Frank et al., 2014; Kim et al., 2017, 2022). The relative effectiveness of the Control training on mental representation structures suggests that in the early stages of perceptual-cognitive scaffolding, physical practice reveals the strongest effects on mental representation development. This is also supported by Rohbanfard and Proteau (2013) who suggest that in AO training conditions interspersed with physical practice, the greater effect of physical practice can “wash-out” the effect of AO, thus overwhelming potential differences between training groups. However, mental representation structures for the AOMI_SELF_ training condition moved further away from the reference structure over time, suggesting the AOMI component of the training disrupted this marker of learning. The AOMI_SELF_ training depicted the novice's own movement technique for the Ankle Pick takedown, providing incorrect visual information about successful execution of the movement that likely developed inaccurate aspects of the mental representation related to sequencing and timing for this motor skill. The provision of this inaccurate visual information, coupled with the novice participants' lack of knowledge about successful execution of the movement, likely resulted in the erroneous formation of aspects of the mental representation related to the sensory consequences for the movement through the MI component of the AOMI_SELF_ training. This is supported by AO literature showing that modeling a novice's own erroneous performance of a motor skill can have detrimental effects on learning (McCullagh et al., 2012). In fact, observing expert performance in isolation or in comparison with novice performance typically results in better long-term retention of complex tasks compared to observing novice performance alone (Andrieux and Proteau, 2013).

This study is the first of its kind to investigate the effects of model type on the effectiveness of AOMI training for novices learning a complex, serial motor skill, but it is important to acknowledge possible limitations associated with the experiment. Firstly, the majority of research investigating the effects of AOMI training on motor skill performance has predominantly focused on simple, discrete motor skills (Chye et al., 2022). To-date, two studies have explored novice learning of complex, serial motor skills in sporting contexts, demonstrating mixed findings across the measures of learning adopted (e.g., Frank et al., 2023; Chye et al., 2024). According to Guadagnoli and Lee's (2004) Challenge-Point framework, motor learning is facilitated when an optimal amount of information is afforded to the learner, with this amount determined by the skill level of the learner as well as the difficulty of the motor skill being learned. The Ankle Pick takedown adopted as the target motor skill in the present study is a highly complex task that includes eight different goal-oriented actions that need to be executed in a coordinated, sequential manner on an opponent. This motor skill was likely too difficult for the novices to learn across the acquisition period adopted in this study (i.e., five training sessions), placing too greater cognitive demands on the learners at this early stage of learning and exceeding their working memory capacity (Furley and Memmert, 2010; Furley and Wood, 2016; Maxwell et al., 2003).

Second, the difficulty of this learning process was likely further increased by the novices performing the Ankle Pick takedown without an opponent during acquisition, and with an opponent during testing sessions. The experiences gained by the novices during acquisition would be used to create estimates to guide their future action (Frank et al., 2024). However, the sensory consequences of this shadow movement differ to those involved with execution of an Ankle Pick takedown on an opponent, due to the altered demands of manipulating and propelling their body. This would increase the cognitive processing demands placed on the novice learners during this testing phase, possibly masking motor learning benefits that may exist for the shadow Ankle Pick takedown that was not assessed during the test phases. In support of this, a previous study by Chye et al. (2024) found that AOMI training of a shadow Osoto Gari judo throw led to improved kinematic measures underpinning successful motor skill performance at the point of retention, indicating that learning of this shadow movement was enhanced through this type of training. This study has greater ecological validity, as it examines if AOMI training of a shadow movement can lead to improved performance of the movement against an opponent, as would be the case in typical sports training or competition. However, there is a need to explore the potential for AOMI training of a complex shadow movement to benefit learning of both the trained shadow movement and a “real world” equivalent, potentially treating the latter as a transfer test (see Schmidt et al., 2018 for a detailed account of transfer and motor skill learning).

Third, the requirements of the motor simulation components of the AOMI training utilized during the acquisition period in this study might not be optimal for novice learners. The AOMI training conditions required the novice learners to alternate between AO of the skeleton-like avatar performing the Ankle Pick takedown from an allocentric and egocentric perspective across consecutive trials. This meant that participants likely engaged in varied levels of mental rotation during the different AOMI trials to support the simultaneous MI of the feeling and sensations involved with performing the movement, increasing the cognitive load placed on participants and potentially reducing the effectiveness of AOMI training (Kim et al., 2022). An asynchronous approach to AOMI training has received support in novices, whereby the learner engages with an AO trial followed by an MI trial during AOMI training (e.g., Romano-Smith et al., 2018, 2019; Kim et al., 2022). The provision of relevant information through AO is likely to facilitate the formation of more accurate mental representations during MI when they are engaged with sequentially (Holmes and Calmels, 2008), especially for novice samples attempting to simulate a complex motor task such as the Ankle Pick takedown utilized in the present study (Kim et al., 2022; Shearer et al., 2009).

This study tested the effects of model type on the effectiveness of AOMI training by comparing self- and other-modeling during AOMI in novices learning a complex grappling takedown. Whilst this approach directly compares two prominent forms of modeling from AO training literature, a large body of work has reported movement benefits after alternative forms of modeling (Ste-Marie et al., 2012, 2020). For example, a mixed-modeling approach where the learner observes both self- and other-models simultaneously or in an alternating fashion, is said to benefit the development of mental representations of movement by providing additional task-related information and distinguishing between successful and unsuccessful movement patterns (Laguna, 2008). Indeed, AO training studies demonstrate greater improvements in motor task performance after mixed-modeling interventions combining a skilled other-model and self-model (e.g., Anderson and Campbell, 2015; Nishizawa and Kimura, 2017; Robertson et al., 2018), learning self-model (Domuracki et al., 2015), and positive self-review model (Barzouka et al., 2015). Future studies should draw from the AO training literature (Ste-Marie et al., 2012, 2020) to comprehensively examine the influence of various combinations of self- and other-model types and model skill level on the effectiveness of AOMI training for motor skill learning across different stages of expertise development and types of motor skill.

In an identical manner to Chye et al. (2024), the AO component of the AOMI training conditions employed in this study displayed 3D-modeled skeleton-like avatars that were digitally generated from whole-body motion capture data. Whilst there is some evidence to suggest that non-realistic human avatars can induce a similar sense of agency as a realistic human avatar (Kim et al., 2020), the propositions of the model-observer similarity hypothesis (Schunk, 1987) indicate that a model that more closely resembles the learner would facilitate the motor learning benefits associated with AOMI training. As such, one possible explanation for the findings of the current study is that the self-model being presented as an anonymous skeleton avatar created a discrepancy in physical similarity between the observer and the avatar, therefore decreasing the level of self-identification the observers had with the self-model avatar, thus confounding potential motor learning benefits. As skilled models are proposedly advantageous in the early stages of learning, particularly when the task to be learned is more complex (Rohbanfard and Proteau, 2011), a method that can combine skilled movement patterns with learner-like resemblance might be optimal (Frank et al., 2024). Recent technologies such as virtual reality and face-swapping make this possible, with studies already showing increased motor system activity (Watanabe et al., 2024) and reduced movement error patterns (Frank et al., 2023) for AOMI employing such techniques. Future research should utilize immersive technologies to further explore AOMI_SELF_ training without being restricted to the performer's skill level. This would allow AOMI training to combine the attentional and motivational benefits of self-modeling with the informational benefits of other-modeling, further enhancing the learning process.

## Conclusion

The present study examined the influence of model type on the effectiveness of AOMI training for novices learning an Ankle Pick takedown. In contrast to our predictions, there was little support for either AOMI_SELF_ or AOMI_OTHER_ training improving motor skill performance compared to the Control training condition, suggesting that physical practice is the primary driver of motor adaptations for this complex motor skill at early learning stages. While there were no significant differences between model types, the AOMI training conditions showed broader reductions in the error scores of discrete kinematic measures of motor skill performance compared to Control training, indicating that an increased training volume may induce greater learning benefits after AOMI training. Self-efficacy levels increased across all training conditions, with the lack of significant differences between conditions suggesting that novice learners primarily derived their self-efficacy beliefs from physical practice of the Ankle Pick takedown. Mental representation structures became more functional in the AOMI_OTHER_ and Control training conditions, with the AOMI_SELF_ training resulting in a less functional mental representation structure at post- and retention-test compared to pre-test. This suggests model type may be an important factor for novices using AOMI training to develop the perceptual-cognitive factors underlying successful motor skill execution. Overall, the findings showed that model type did not have an influence on the effectiveness of AOMI interventions to improve learning of a complex motor skill in novices for the present study, suggesting the need to empirically investigate the effectiveness of mixed-modeling approaches that incorporate both self- and other-modeling when utilizing AOMI training for this purpose in sport.

## Data Availability

The datasets presented in this study can be found in online repositories. The names of the repository/repositories and accession number(s) can be found below: https://osf.io/3qgty/.
