# Supplementary material for: Does model type influence the effectiveness of combined action observation and motor imagery training for novices learning an Ankle Pick takedown?
Source: Front Psychol. 2025 Jun 17;16:1596660. doi: 10.3389/fpsyg.2025.1596660 (PMC12209306; doi:10.3389/fpsyg.2025.1596660)
Supplement: Supplementary file 1 [file Supplementary_file_1.docx]

**Supplementary Results**

**Table S1. Random effect residuals for participant across the MLMs for the different outcome measures recorded to infer learning in this study**

| Variable | τ00 Participant | Variance |
| --- | --- | --- |
| Base of Support | 43.87 | 39.19% |
| Horizontal Centre of Mass | 60.36 | 77.02% |
| Vertical Centre of Mass | 74.14 | 71.3% |
| Left Hip Angle | 87.47 | 35.51% |
| Left Knee Angle | 193.6 | 49.27% |
| Time Difference | 0.01626 | 48.77% |
| Change Y | 0.0007496 | 26.89% |
| Change Z | 79.30 | 68.84% |
| Average Velocity | 0.002411 | 13.14% |
| Peak Right Knee Velocity | 336.7 | 37.55% |
| Peak Right Ankle Velocity | 1893 | 38.62% |
| Peak Vertical Velocity | 0.01248 | 68.57% |
| Self-Efficacy Score | 1.6860 | 73.46% |

**Exploring Imagery Ability as a Co-Variate**

***Motor Skill Performance***

**Base of Support.** The MLM incorporating imagery ability as a co-variate increased the accuracy of the model (*BIC* = 653.6) compared to the original (*BIC* = 649.4), and imagery ability scores did not significantly influence base of support error scores (*ß =* 0.04, *p* = .67).

**Horizontal Centre of Mass.** The MLM incorporating imagery ability as a co-variate increased the accuracy of the model (*BIC* = 514.7) compared to the original (*BIC* = 512), but imagery ability scores did not significantly influence horizontal centre of mass error scores (*ß =* -0.12, *p* = .21).

**Vertical Centre of Mass.** The MLM incorporating imagery ability as a co-variate increased the accuracy of the model (*BIC* = 633.7) compared to the original (*BIC* = 630.3), but imagery ability scores did not significantly influence vertical centre of mass error scores (*ß =* -0.11, *p* = .3).

**Left Hip Angle.** The MLM incorporating imagery ability as a co-variate increased the accuracy of the model (*BIC* = 718.5) compared to the original (*BIC* = 715.2), but imagery ability scores did not significantly influence left hip angle error scores (*ß =* 0.15, *p* = .28).

**Left Knee Angle.** The MLM incorporating imagery ability as a co-variate increased the accuracy of the model (*BIC* = 746.5) compared to the original (*BIC* = 743.9), but imagery ability scores did not significantly influence left knee angle error scores (*ß =* -0.24, *p* = .18).

**Time Difference.** The MLM incorporating imagery ability as a co-variate decreased the accuracy of the model (*BIC* = -23.2) compared to the original (*BIC* = -17), but imagery ability scores did not significantly influence time difference error scores (*ß =* -0.0003, *p* = .85).

**Displacement of horizontal centre of mass.** The MLM incorporating imagery ability as a co-variate increased the accuracy of the model (*BIC* = -187.4) compared to the original (*BIC* = -191.5), but imagery ability scores did not significantly influence displacement of horizontal centre of mass error scores (*ß =* 0.0002, *p* = .66).

**Displacement of vertical centre of mass.** The MLM incorporating imagery ability as a co-variate increased the accuracy of the model (*BIC* = 648.1) compared to the original (*BIC* = 644.8), but imagery ability scores did not significantly displacement of vertical centre of mass error scores (*ß =* -0.11, *p* = .31).

**Average Velocity during lowering phase.** The MLM incorporating imagery ability as a co-variate increased the accuracy of the model (*BIC* = -40.5) compared to the original (*BIC* = -44.8), but imagery ability scores did not significantly influence average velocity during lowering phase error scores (*ß =* 0.0002, *p* = .81).

**Peak Right Knee Angle Velocity.** The MLM incorporating imagery ability as a co-variate decreased the accuracy of the model (*BIC* = 817.7) compared to the original (*BIC* = 818.9), but imagery ability scores did significantly influence peak right knee angle velocity error scores (*ß =* -0.62, *p* = .02).

**Peak Right Ankle Angle Velocity.** The MLM incorporating imagery ability as a co-variate decreased the accuracy of the model (*BIC* = 945.5) compared to the original (*BIC* = 955.8), but imagery ability scores did significantly influence peak right ankle angle velocity error scores (*ß =* -2.03, *p* <.001).

**Peak Vertical Velocity.** The MLM incorporating imagery ability as a co-variate increased the accuracy of the model (*BIC* = -85.8) compared to the original (*BIC* = -86.5), but imagery ability scores did not significantly influence peak vertical velocity error scores (*ß =* 0.003, *p* = .06).

**Self-Efficacy Score.** The MLM incorporating imagery ability as a co-variate increased the accuracy of the model (*BIC* = 311.7) compared to the original (*BIC* = -307.9), but imagery ability scores did not significantly influence peak vertical velocity error scores (*ß =* -0.01, *p* = .43).

**Social Validation Data**

Participants allocated to the two AOMI groups responded positively to the social validation questionnaire (Table _) with a mean rating of 1.58 recorded for the AOMI_OTHER_ training condition and 1.86 recorded for the AOMI_SELF_ training condition. There were no significant differences between the two training conditions. Below are summary responses and representative quotes for the effects of the different AOMI training conditions on the measures of learning, as well as the differences in their perceptions of the avatars.

***Motor Skill Performance***

All ten participants (100%) believed that their motor skill performance improved after both AOMI_OTHER_ and AOMI_SELF_ training. The participants that perceived AOMI_OTHER_ training as performance enhancing suggested that the AOMI stimuli provided useful visual information and technical detail about performing the ankle pick movement; *P13 “when I looked at the videos of the avatar, I could see where I needed to place my body, so over time, as I saw them more and more, I was watching the videos and I could see more and more detail”.* The participants that perceived AOMI_SELF_ training as performance enhancing suggested that the AOMI stimuli facilitated a positive sense of self and provided opportunities for error detection and correction in performing the movement; *P18 “it was more like a reassurance of how to do it, like a revision. And when I did the movement, I observed whatever the avatar did and it kind of had an impact on how I did it…Along the course I remember correcting a few things that I had missed earlier so I think that definitely impacted the performance of the movement”.*

**Self-Efficacy**

Nine participants (90%) believed that their self-efficacy increased after AOMI_OTHER_ and all ten participants (100%) believed that their self-efficacy increased after AOMI_SELF_ training. The participants that perceived AOMI_OTHER_ training to have a positive impact on their self-efficacy suggested that the videos facilitated the provision of positive mastery experiences; *P15 “Because watching, and watching and doing and to practice, it just made me feel more confident that what I was doing was right as opposed to negatively impacting me”.* Similarly, participants that perceived AOMI_SELF_ training to have a positive impact on their self-efficacy also suggested that the videos facilitated the provision of positive mastery experiences; P18 *“Because by watching it, it made me realise what it involves, so therefore I could analyse and think whether I could be able to do it and it seemed like it was something that I could do, therefore I felt more confident”.*

**Mental Representation Structure**

Nine participants (90%) believed that their understanding of the movement improved after AOMI_OTHER_ training and all ten participants (100%) believed that their understanding of the movement improved after AOMI_SELF_ training. The participants that perceived AOMI_OTHER_ training to advance their knowledge about the ankle pick suggested it provided useful cues such as spatial information that helped them to further their understanding of the movement; *P17 “looking at it and thinking about it, it gave me a full understanding of the whole movement because I think when you looked at it either from the front view or just above the head, you knew where you were in space and how to do the movement”.* The participants that perceived AOMI_SELF_ training to be beneficial in developing their understanding of the movement suggested that it helped them to consolidate different aspects of the movement to form a better understanding of it overall; *P25 “I can almost focus on a different part of the skeleton each time I watched it, so rather than looking at the whole thing, I can be like I’m going to see what the legs are doing so you kind of learn something about the movement every time you watch it”.*

**Table S2. Social validation questionnaire responses for the AOMI training conditions.**

| Social Validation Questionnaire Item | AOMI_OTHER_ | AOMI_SELF_ | Diff |
| --- | --- | --- | --- |
|  | ***n = 10*** | ***n = 10*** | ***t-statistic*** |
| Motor Skill Performance^a^ – perceived impact of AOMI training on performance of Osoto Gari. | 3 ± 0.94 | 2.9 ± 1.2 | 0.1 |
| Self-Efficacy^a^ - perceived impact of AOMI training on confidence to perform Osoto Gari successfully. | 2.8 ± 1.62 | 3.1 ± 1.37 | -0.3 |
| Mental Representation Structure^a^ - perceived impact of AOMI training on understanding of the Osoto Gari. | 2.7 ± 2.41 | 3.5 ± 1.08 | -0.8 |
| AO Ability^b^ – ability to watch the Osoto Gari videos during the AOMI training. | 1.1 ± 1.29 | 1.8 ± 1.4 | -0.7 |
| MI Ability^b^ - ability to imagine the sensations involved with the Osoto Gari during the AOMI training. | 0.9 ± 1.66 | 1.3 ± 1.06 | -0.4 |
| AOMI Difficulty^b^ - ability to simultaneously watch the videos and imagine the sensations involved with the Osoto Gari during the AOMI training. | 0.5 ± 1.43 | 0.3 ± 1.64 | 0.2 |
| MI Vividness^c^ – clarity and vividness of the sensations generated when imagining the Osoto Gari during the AOMI training. | 1.1 ± 0.99 | 0.6 ± 1.43 | 0.5 |
| Avatar Representativeness^d^ – perceived similarity between the participant’s Osoto Gari and the avatar’s performance in the videos during AOMI training. | 1.6 ± 1.17 | 1.8 ± 0.79 | -0.2 |
| Avatar Plausibility^d^ – perceived plausibility of the Osoto Gari performed by the avatar in the videos during AOMI training. | 1.5 ± 0.71 | 2.1 ± 0.88 | -0.6 |
| Avatar Ownership^d^ - perceived ownership of the Osoto Gari performed by the avatar in the videos during AOMI training. | 0.6 ± 1.84 | 1.2 ± 1.75 | -0.6 |

*Note. The following rating scales were adopted across the questionnaire: ^a^ -5 (negative effect) to 5 (positive effect); ^b^ -3 (very difficult) to 3 (very easy); ^c^ -3 (very unclear and not at all vivid) to 3 (very clear and vivid); ^d^ -3 (strongly disagree) to 3 (strongly agree). *p < .05*
